# Processes of Care Associated With Risk of Mortality and Recurrent Stroke Among Patients With Transient Ischemic Attack and Nonsevere Ischemic Stroke

**DOI:** 10.1001/jamanetworkopen.2019.6716

**Published:** 2019-07-03

**Authors:** Dawn M. Bravata, Laura J. Myers, Mathew Reeves, Eric M. Cheng, Fitsum Baye, Susan Ofner, Edward J. Miech, Teresa Damush, Jason J. Sico, Alan Zillich, Michael Phipps, Linda S. Williams, Seemant Chaturvedi, Jason Johanning, Zhangsheng Yu, Anthony J. Perkins, Ying Zhang, Greg Arling

**Affiliations:** 1 Veterans Affairs Health Services Research and Development, Precision Monitoring to Transform Care, Quality Enhancement Research Initiative, Department of Veterans Affairs, Indianapolis, Indiana; 2 Veterans Affairs Health Services Research and Development, Center for Health Information and Communication, Richard L. Roudebush VA Medical Center, Indianapolis, Indiana; 3 Department of Internal Medicine, Indiana University School of Medicine, Indianapolis; 4 Regenstrief Institute, Indianapolis, Indiana; 5 Department of Epidemiology, Michigan State University, East Lansing; 6 Department of Neurology, VA Greater Los Angeles Healthcare System, Los Angeles, California; 7 Department of Neurology, David Geffen School of Medicine, University of California, Los Angeles; 8 Department of Biostatistics, Indiana University School of Medicine, Indianapolis; 9 Department of Internal Medicine, Yale University School of Medicine, New Haven, Connecticut; 10 Department of Neurology, Yale University School of Medicine, New Haven, Connecticut; 11 Clinical Epidemiology Research Center, VA Connecticut Healthcare System, West Haven; 12 Department of Pharmacy Practice, Purdue University College of Pharmacy, West Lafayette, Indiana; 13 Department of Neurology, University of Maryland School of Medicine, Baltimore; 14 Department of Neurology, Indiana University School of Medicine, Indianapolis; 15 Omaha Division, VA Nebraska-Western Iowa Health Care System, Omaha; 16 Department of Surgery, University of Nebraska, Lincoln; 17 Purdue University School of Nursing, Lafayette, Indiana

## Abstract

**Importance:**

Early evaluation and management of patients with transient ischemic attack (TIA) and nonsevere ischemic stroke improves outcomes.

**Objective:**

To identify processes of care associated with reduced risk of death or recurrent stroke among patients with TIA or nonsevere ischemic stroke.

**Design, Setting, and Participants:**

This cohort study included all patients with TIA or nonsevere ischemic stroke at Department of Veterans Affairs emergency department or inpatient settings from October 2010 to September 2011. Multivariable logistic regression was used to model associations of processes of care and without-fail care, defined as receiving all guideline-concordant processes of care for which patients are eligible, with risk of death and recurrent stroke. Data were analyzed from March 2018 to April 2019.

**Main Outcomes and Measures:**

Risk of all-cause mortality and recurrent ischemic stroke at 90 days and 1 year was calculated. Overall, 28 processes of care were examined. Without-fail care was assessed for 6 processes: brain imaging, carotid artery imaging, hypertension medication intensification, high- or moderate-potency statin therapy, antithrombotics, and anticoagulation for atrial fibrillation.

**Results:**

Among 8076 patients, the mean (SD) age was 67.8 (11.6) years, 7752 patients (96.0%) were men, 5929 (73.4%) were white, 474 (6.1%) had a recurrent ischemic stroke within 90 days, 793 (10.7%) had a recurrent ischemic stroke within 1 year, 320 (4.0%) died within 90 days, and 814 (10.1%) died within 1 year. Overall, 9 processes were independently associated with lower odds of both 90-day and 1-year mortality after adjustment for multiple comparisons: carotid artery imaging (90-day adjusted odds ratio [aOR], 0.49; 95% CI, 0.38-0.63; 1-year aOR, 0.61; 95% CI, 0.52-0.72), antihypertensive medication class (90-day aOR, 0.58; 95% CI, 0.45-0.74; 1-year aOR, 0.70; 95% CI, 0.60-0.83), lipid measurement (90-day aOR, 0.68; 95% CI, 0.51-0.90; 1-year aOR, 0.64; 95% CI, 0.53-0.78), lipid management (90-day aOR, 0.46; 95% CI, 0.33-0.65; 1-year aOR, 0.67; 95% CI, 0.53-0.85), discharged receiving statin medication (90-day aOR, 0.51; 95% CI, 0.36-0.73; 1-year aOR, 0.70; 95% CI, 0.55-0.88), cholesterol-lowering medication intensification (90-day aOR, 0.47; 95% CI, 0.26-0.83; 1-year aOR, 0.56; 95% CI, 0.41-0.77), antithrombotics by day 2 (90-day aOR, 0.56; 95% CI, 0.40-0.79; 1-year aOR, 0.69; 95% CI, 0.55-0.87) or at discharge (90-day aOR, 0.59; 95% CI, 0.41-0.86; 1-year aOR, 0.69; 95% CI, 0.54-0.88), and neurology consultation (90-day aOR, 0.67; 95% CI, 0.52-0.87; 1-year aOR, 0.74; 95% CI, 0.63-0.87). Anticoagulation for atrial fibrillation was associated with lower odds of 1-year mortality only (aOR, 0.59; 95% CI, 0.40-0.85). No processes were associated with reduced risk of recurrent stroke after adjustment for multiple comparisons. The rate of without-fail care was 15.3%; 1216 patients received all guideline-concordant processes of care for which they were eligible. Without-fail care was associated with a 31.2% lower odds of 1-year mortality (aOR, 0.69; 95% CI, 0.55-0.87) but was not independently associated with stroke risk.

**Conclusions and Relevance:**

Patients who received 6 readily available processes of care had lower adjusted mortality 1 year after TIA or nonsevere ischemic stroke. Clinicians caring for patients with TIA and nonsevere ischemic stroke should seek to ensure that patients receive all guideline-concordant processes of care for which they are eligible.

## Key Points

**Question:**

Which processes of care are associated with reduced risk of mortality or recurrent stroke after transient ischemic attack or nonsevere ischemic stroke?

**Findings:**

In this cohort study of 8076 patients with transient ischemic attack or nonsevere ischemic stroke, only 1216 (15.3%) received without-fail care, defined as receiving all guideline-concordant processes of care for which they were eligible (ie, brain imaging, carotid artery imaging, antihypertensive intensification, high- or moderate-potency statin therapy, antithrombotics, and anticoagulation for atrial fibrillation). Receiving all 6 processes was associated with lower risk of death (31.2% reduction at 1 year) but not lower risk of recurrent stroke.

**Meaning:**

Clinicians should ensure that patients with transient ischemic attack and nonsevere ischemic stroke receive all guideline-concordant processes of care for which they are eligible.

## Teaser

This cohort study identifies processes of care that are associated with reduced risk of death or recurrent stroke among patients with transient ischemic attack or nonsevere ischemic stroke.

## Introduction

Patients with transient ischemic attack (TIA) and nonsevere ischemic stroke are at high risk of recurrent vascular events.^1,2,3^ However, studies have demonstrated that timely delivery of guideline-concordant care can dramatically reduce this risk.^4,5,6,7^ Studies reporting risk reductions of at least 70% for recurrent events among patients with TIA or nonsevere ischemic stroke have emphasized early evaluation and management; however, these studies differed in terms of the processes of care that were provided.^4-8^ The American Heart Association/American Stroke Association (AHA/ASA) stroke prevention guidelines recommend a broad range of processes, including diagnostic processes (eg, brain imaging) and secondary prevention interventions (eg, hypertension management).^8^ Our objective was to identify the guideline-concordant processes of care that were associated with a reduction in the risk of recurrent ischemic stroke or death among patients with TIA or nonsevere stroke, adjusting for baseline patient characteristics.

## Methods

This study follows the Strengthening the Reporting of Observational Studies in Epidemiology (STROBE) reporting guideline. Human subjects approvals were obtained from the Indiana University School of Medicine Institutional Review Board and the Richard L. Roudebush VA Medical Center Research and Development Committee. Given that data were obtained from administrative sources, we obtained a waiver of written informed consent.

### Cohort Construction

We identified patients with TIA or ischemic stroke (minor or major) cared for in Department of Veterans Affairs (VA) emergency departments or inpatient settings in fiscal year 2011 (October 2010 to September 2011) using primary discharge codes for TIA (*International Classification of Diseases, Ninth Revision *[*ICD*-*9*] codes: 435.0, 435.1, 435.3, 435.8, and 435.9) or ischemic stroke (*ICD*-*9 *codes: 433.X1, 434.00, 434.X1, and 436).^9,10^ Electronic health record data did not include a measure of stroke severity; therefore, we used a validated approach to identify and exclude patients with major stroke (ie, length of stay >6 days, ventilator use, feeding tube use, coma, intensive care unit stay, inpatient rehabilitation stay, or thrombolysis).^10^ Patients transferred to non–Veterans Health Administration (VHA) facilities were excluded because we could not assess quality of care.

### Outcomes

The 4 primary outcomes were 90-day and 1-year risk of all-cause mortality and recurrent ischemic stroke.^11^ We chose a 90-day end point because this timeframe has been used in TIA and nonsevere ischemic stroke studies^4,5,7^ and a 1 year end point because some processes (eg, hypertension control) are thought to improve outcomes during longer time horizons. Ischemic stroke outcome events included emergency department or inpatient visits with a diagnosis code used for cohort construction. Ischemic stroke events occurring the day after discharge from the index event were not considered recurrent because medical record review indicated that the second visit was nearly always a continuation of care for the index event. All patients were included in the mortality outcomes. Patients transferred to non-VHA facilities or who died during the index event were excluded from recurrent stroke outcomes.

### Processes of Care

Overall, 28 processes of care were evaluated using electronic quality measures (eQMs) previously validated against medical record review.^10,12^ The processes were AHA/ASA recommended elements of care^8^; some were similar to The Joint Commission stroke core measure set processes^13^ and some were in the AHA/ASA Performance Measures Set.^14^ To our knowledge, neither organization has a TIA-specific measure set. Numerator, denominator, and exclusion definitions are in eTable 1 in the Supplement. The processes included diagnostic and therapeutic components of care: carotid artery imaging; carotid stenosis procedure (ie, endarterectomy or stent); antihypertensive medication intensification (ie, new medication or increased dosage of existing medication); hypertension control (ie, mean blood pressure <140/90 mm Hg in the 90 days after discharge); antihypertensive medication class^15^ (ie, angiotensin-converting enzyme inhibitor or angiotensin receptor blocker for patients with chronic kidney disease; thiazide for black patients; thiazide, angiotensin-converting enzyme inhibitor, or angiotensin receptor blocker for others); lipid measurement; lipid management (ie, cholesterol-reducing medication if low-density lipoprotein cholesterol [LDL-C] levels ≥100 mg/dL [to convert millimoles per liter, multiply by 0.0259] or no measurement or if LDL-C <100 mg/dL or missing but patient is using existing lipid-lowering medication); discharged receiving statin medication; cholesterol-lowering medication intensification (ie, if LDL-C ≥100 mg/dL or no measurement among patients on submaximal lipid-lowering regimen); high- or moderate-potency statin prescription (ie, high-potency statin if patient is aged <75 years; moderate- or high-potency statin if patient is aged ≥75 years); oral hypoglycemic medication intensification; hemoglobin A_1c_ measurement; electrocardiography; telemetry; Holter monitor use; antithrombotics at day 2; antithrombotics at discharge; anticoagulation for atrial fibrillation or flutter; international normalized ratio (INR) measurement; anticoagulation quality (ie, INR 2-3 within 30 days of discharge); brain imaging; deep vein thrombosis prophylaxis; rehabilitation needs assessment; speech-language pathology consultation; substance-use treatment referral for patients with alcohol use disorder; nicotine replacement therapy for smokers; polysomnography; and neurology consultation.

For each process, patients were classified as ineligible, eligible and passed, or eligible and failed. The carotid stenosis measure was assessed in the 14 days after presentation; patients with an outcome event in the 14 days after presentation were excluded. For measures assessed during 30 days after discharge, patients with outcomes prior to the 30-day mark were excluded. For the 2 measures assessed during 90 days after discharge (ie, hypertension control and polysomnography), we reported outcomes at 1 year and excluded patients with events prior to 90 days.

A variety of data sources were used to construct the eQMs.^10^ Veterans Health Administration Central Data Warehouse inpatient and outpatient data files in the 5 years before the event were used to identify medical history, health care utilization, and procedures (using *Current Procedural Terminology*, Healthcare Common Procedures Coding System, and *ICD*-*9* procedure codes).^16^ Pharmacy Benefits Management data were used to identify medications. Data from the VHA Central Data Warehouse were also used for vital signs, laboratory data, allergies, orders, and consultations. Linked VA/US Centers for Medicare & Medicaid Services data were used to identify hospitalizations in non-VHA facilities. Veterans Affairs Fee Basis data were used to identify inpatient and outpatient use and medical history. The date of death was obtained from VA Vital Status files.

### Without-Fail Care

Our goal was to identify the processes of care that were associated with improvements in vascular risk and could be implemented across health care systems. It might not be possible to identify individual processes that confer benefit because patients may receive several processes at the same time or through a shared structure of care (eg, an admission order set). Although the VA system does not use a specific TIA or stroke admission order set or care pathway, we hypothesized that a bundle of processes might be routinely ordered, and therefore, we examined the 6 processes that were found to be effective in acute TIA management studies (ie, brain imaging, carotid artery imaging, hypertension medication intensification, high- or moderate-potency statin therapy, antithrombotics, and anticoagulation for atrial fibrillation).^6^ The 6 without-fail processes should be routinely available because they do not require advanced structures of care. A without-fail care rate^17,18,19^ was calculated for patients who were eligible for at least 1 of the 6 processes. Patients who received all without-fail care processes for which they were eligible were classified as passing the without-fail care rate.

### Statistical Analysis

Patient characteristics, medical history, concomitant comorbidities, the final index event diagnosis (TIA or nonsevere ischemic stroke), pre–index event inpatient and outpatient use, and index event symptoms were compared among patients with outcomes and patients without outcomes. Continuous variables were summarized using means and SDs or medians and ranges. The Wilcoxon rank sum test was used to test differences in continuous variables, and Fisher exact or χ^2^ tests were used to compare categorical variables between groups.

Multivariable modeling was conducted in 2 phases for each of the 4 outcomes. First, we constructed risk-adjustment models and then used the risk-adjustment models to assess associations of processes with outcomes. First, 4 logistic regression models with random facility effects (to account for clustering of patients within facilities) were used to model the outcomes. Independent variables based on patient and facility factors (but not eQMs) were divided into 2 types: those which clinical judgment deemed a priori confounders were forced into the model and those eligible for potential inclusion in the final model based on backward variable selection (*P* < .25 to enter, *P* < .05 to stay). All tests were 2-tailed.

Second, using the models built in the first phase, the eQMs were included individually as covariates by creating 2 indicator variables for each process: (1) passing among eligible patients and (2) not eligible. The reference category was being eligible and failing; therefore, the results can be interpreted as passing vs failing. Including patients who were not eligible allowed all cohort members to be included in each model and facilitated our identification of processes that were most closely associated with outcomes. Each eQM was evaluated in a separate risk-adjusted model that did not include other eQMs. To enhance the clarity of tables, we present odds ratios (ORs), 95% CIs, and *P* values for passing the process with failing as the reference (data for ineligible patients are in eTables 2-5 in the Supplement). Statistical significance was set at *P* < .05, and tests were 2-tailed. We adjusted for multiple comparisons using the false discovery rate within each domain of interest (each process and the without-fail rate).^20^

We conducted 3 sensitivity analyses. First, we repeated the modeling with patients 65 years or older because Centers for Medicare & Medicaid Services data for this population augments outcome data available from VA sources (ie, hospitalizations for ischemic stroke at non-VHA facilities that were not paid for by the VA). Second, we examined the outcomes of quality of care for patients with TIA vs nonsevere ischemic stroke. Third, we examined 3 medication measures to evaluate whether prior medication use (eg, being prescribed a statin before the index TIA or nonsevere ischemic stroke event) accounted for the association of processes with outcomes. Patients who passed each medication measure were classified into 2 groups: passed and taking medication before the index event vs passed and taking new medication after the index event. These 2 variables were entered in the final logistic regression models (the reference group included patients who failed the measure). Data analyses were performed using SAS software version 9.2 (SAS Institute).

## Results

Among 8076 patients, 3863 (47.8%) had TIA and 4213 (52.2%) had nonsevere ischemic stroke. A total of 474 of 7802 at risk (6.1%) had a recurrent stroke within 90 days, and 793 of 7387 at risk (10.7%) had a recurrent stroke within 1 year; 320 of 8076 (4.0%) died within 90 days, and 814 of 8076 (10.1%) died within 1 year. The mean (SD) age was 67.8 (11.6) years, 7752 (96.0%) were men, and 5929 (73.4%) were white (Table 1). The multivariable risk-adjustment models are provided in Table 2. The final risk-adjustment model included 25 patient-level characteristics (included in Table 1) and no facility-level characteristics. The C statistics for the risk-adjustment models were 0.815 for 90-day mortality, 0.789 for 1-year mortality, 0.657 for 90-day recurrent stroke, and 0.659 for 1-year recurrent stroke.

**Table 1.** Baseline Characteristics for 8076 Patients with TIA or Nonsevere Stroke

Characteristic	No. (%)
Age, y	
Mean (SD)	67.8 (11.6)
Median (IQR) [range]	65 (60-77) [20-101]
Men	7752 (96.0)
Race	
White	5929 (73.4)
Black	1726 (21.4)
Other	421 (5.2)
Stroke in prior 30 d	764 (9.5)
TIA in prior 30 d	312 (3.9)
Carotid artery stenosis	1294 (16.0)
Carotid endarterectomy or stent	544 (6.7)
Diabetes	3320 (41.1)
Hypertension	6549 (81.1)
Hyperlipidemia	5824 (72.1)
Congestive heart failure	1859 (23.0)
Myocardial infarction, CABG, or PCI/stent	2819 (34.9)
Atrial fibrillation	1077 (13.3)
Other cardiac arrhythmia	2150 (26.6)
Peripheral arterial disease	2244 (27.8)
Chronic kidney disease	1549 (19.2)
Charlson Comorbidity Index score, median (IQR) [range]	1 (0-3) [0-18]
Concomitant medical conditions	
Congestive heart failure, ie, BNP >200 pg/mL	84 (1.0)
Myocardial infarction, ie, troponin >0.1 ng/mL	22 (0.3)
Current tobacco smoker	2748 (34.0)
Index event diagnosis	
TIA	3863 (47.8)
Minor ischemic stoke	4213 (52.2)

Abbreviations: BNP, B-type natriuretic peptide; CABG, coronary artery bypass grafting; IQR, interquartile range; PCI, percutaneous coronary intervention; TIA, transient ischemic attack.

SI conversion factors: To convert BNP to nanograms per liter, multiply by 1.0; troponin to micrograms per liter, multiply by 1.0.

**Table 2.** Final Risk-Adjustment Model for 90-Day and 1-Year Mortality and Recurrent Ischemic Stroke Events

Patient Characteristic	90-d Outcomes				1-y Outcomes			
	Mortality		Recurrent Ischemic Stroke		Mortality		Recurrent Ischemic Stroke	
	OR (95% CI)	*P* Value	OR (95% CI)	*P* Value	OR (95% CI)	*P* Value	OR (95% CI)	*P* Value
No./total No. (%)	320/8076 (4.0)		474/7802 (6.1)		814/8076 (10.1)		793/7387 (10.7)	
Age	1.06 (1.05-1.07)	<.001	1.00 (0.99-1.01)	.31	1.06 (1.05-1.07)	<.001	1.00 (0.99-1.01)	.42
Female sex	0.93 (0.42-2.05)	.86	0.43 (0.21-0.87)	.02	0.71 (0.41-1.23)	.22	0.77 (0.50-1.20)	.26
Race								
Black	0.92 (0.66-1.26)	.59	0.99 (0.78-1.26)	.96	0.87 (0.71-1.08)	.22	1.08 (0.90-1.30)	.42
Other	0.62 (0.31-1.25)	.18	0.86 (0.54-1.35)	.50	1.00 (0.69-1.46)	>.99	1.00 (0.70-1.42)	>.99
Stroke in prior 30 d	1.26 (0.89-1.77)	.19	1.56 (1.18-2.07)	.002	1.06 (0.83-1.36)	.64	1.58 (1.26-1.98)	<.001
TIA in prior 30 d prior to index event	1.02 (0.56-1.86)	.95	0.87 (0.52-1.45)	.60	0.96 (0.65-1.43)	.86	0.92 (0.62-1.37)	.69
Carotid artery stenosis	0.80 (0.59-1.10)	.17	1.31 (1.01-1.70)	.04	0.90 (0.73-1.11)	.32	1.30 (1.06-1.60)	.01
Carotid endarterectomy or stent	0.55 (0.31-0.97)	.04	0.92 (0.63-1.34)	.66	0.83 (0.60-1.14)	.24	0.91 (0.67-1.23)	.53
Diabetes	0.78 (0.60-1.02)	.07	1.19 (0.96-1.49)	.12	0.83 (0.70-1.00)	.05	1.11 (0.94-1.33)	.23
Hypertension	1.07 (0.70-1.62)	.76	0.92 (0.70-1.22)	.56	0.87 (0.67-1.13)	.30	1.02 (0.81-1.28)	.90
Hyperlipidemia	0.70 (0.52-0.95)	.02	0.97 (0.76-1.23)	.80	0.75 (0.61-0.92)	.006	0.89 (0.73-1.08)	.23
Congestive heart failure	1.17 (0.88-1.56)	.29	0.93 (0.72-1.21)	.60	1.10 (0.91-1.34)	.32	1.00 (0.81-1.23)	>.99
Myocardial infarction, CABG, or PCI/stent	1.06 (0.80-1.39)	.70	1.15 (0.92-1.45)	.22	1.08 (0.90-1.30)	.43	1.15 (0.95-1.38)	.15
Atrial fibrillation	1.66 (1.24-2.21)	.001	1.23 (0.91-1.66)	.17	1.55 (1.27-1.90)	<.001	1.18 (0.93-1.50)	.18
Other cardiac arrhythmia	1.02 (0.78-1.35)	.87	0.86 (0.67-1.09)	.21	0.85 (0.71-1.03)	.09	0.86 (0.71-1.04)	.12
Peripheral arterial disease	1.47 (1.13-1.91)	.004	1.07 (0.85-1.35)	.56	1.28 (1.07-1.52)	.007	1.15 (0.96-1.38)	.13
Chronic kidney disease	0.62 (0.46-0.85)	.003	0.97 (0.73-1.28)	.82	0.82 (0.67-1.01)	.06	0.96 (0.77-1.19)	.72
Charlson Comorbidity Index score	1.22 (1.16-1.29)	<.001	1.01 (0.95-1.07)	.82	1.23 (1.18-1.28)	<.001	1.08 (1.03-1.13)	.001
Congestive heart failure, ie, BNP >200 pg/mL	2.12 (1.08-4.15)	.03	0.19 (0.03-1.40)	.10	2.14 (1.28-3.57)	.004	0.11 (0.02-0.77)	.03
Myocardial infarction, ie, troponin >0.1 ng/mL	5.36 (1.73-16.65)	.004	7.26 (2.65-19.89)	<.001	4.00 (1.51-10.60)	.005	3.98 (1.43-11.05)	.008
Current tobacco smoker	1.19 (0.89-1.58)	.24	1.03 (0.84-1.27)	.78	1.14 (0.95-1.38)	.16	1.00 (0.84-1.18)	.96
Index event diagnosis of stroke	2.09 (1.62-2.69)	<.001	2.00 (1.63-2.45)	<.001	1.55 (1.32-1.82)	<.001	1.77 (1.51-2.08)	<.001
No. of hospitalizations pre–index event	1.06 (0.99-1.13)	.07	1.08 (1.02-1.15)	.008	1.11 (1.06-1.17)	<.001	1.07 (1.02-1.13)	.01
Speech deficit	1.32 (0.99-1.76)	.06	1.31 (1.03-1.66)	.03	1.26 (1.03-1.53)	.02	1.21 (1.00-1.47)	.06
APACHE score	1.05 (1.03-1.06)	<.001	1.00 (0.98-1.01)	.75	1.04 (1.02-1.05)	<.001	1.02 (1.00-1.03)	.01

Abbreviations: APACHE, Acute Physiology and Chronic Health Evaluation; BNP, B-type natriuretic peptide; CABG, coronary artery bypass grafting; OR, odds ratio; PCI, percutaneous coronary intervention; TIA, transient ischemic attack.

SI conversion factors: To convert BNP to nanograms per liter, multiply by 1.0; troponin to micrograms per liter, multiply by 1.0.

The associations of individual processes with outcomes are shown in Table 3. Overall, 9 processes were independently associated with lower odds of 90-day mortality after adjustment for multiple comparisons: carotid artery imaging (adjusted OR [aOR], 0.49; 95% CI, 0.38-0.63), antihypertensive medication class (aOR, 0.58; 95% CI, 0.45-0.74), lipid measurement (aOR, 0.68; 95% CI, 0.51-0.90), lipid management (aOR, 0.46; 95% CI, 0.33-0.65), discharged receiving statin medication (aOR, 0.51; 95% CI, 0.36-0.73), cholesterol-lowering medication intensification (aOR, 0.47; 95% CI, 0.26-0.83), antithrombotics by day 2 (aOR, 0.56; 95% CI, 0.40-0.79), antithrombotics at discharge (aOR, 0.59; 95% CI, 0.41-0.86), and neurology consultation (aOR, 0.67; 95% CI, 0.52-0.87). For 1-year mortality, 10 processes were independently associated with lower risk after adjustment for multiple comparisons: carotid artery imaging (aOR, 0.61; 95% CI, 0.52-0.72), antihypertensive medication class (aOR, 0.70; 95% CI, 0.60-0.83), lipid measurement (aOR, 0.64; 95% CI, 0.53-0.78), lipid management (aOR, 0.67; 95% CI, 0.53-0.85), discharged receiving statin medication (aOR, 0.70; 95% CI, 0.55-0.88), cholesterol-lowering medication intensification (aOR, 0.56; 95% CI, 0.41-0.77), antithrombotics by day 2 (aOR, 0.69; 95% CI, 0.55-0.87), antithrombotics at discharge (aOR, 0.69; 95% CI, 0.54-0.88), anticoagulation for atrial fibrillation (aOR, 0.59; 95% CI, 0.40-0.85), and neurology consultation (aOR, 0.74; 95% CI, 0.63-0.87). No process was independently associated with recurrent stroke outcomes after adjustment for multiple comparisons. The pass rates for the processes and complete model results are provided in eTables 2-5 in the Supplement; raw *P* values and *P* values adjusted for multiple comparisons are provided in eTable 8 in the Supplement.

**Table 3.** Risk-Adjusted Association of Passing an Individual Process of Care Measure With Recurrent Vascular Events

Process Measures	Risk-Adjusted OR (95% CI)^a^			
	90-d Outcomes		1-y Outcomes	
	Mortality Risk	Recurrent Stroke Risk	Mortality Risk	Recurrent Stroke Risk
No./total No. (%)	320/8076 (4.0)	474/7802 (6.1)	814/8076 (10.1)	793/7387 (10.7)
Carotid				
Carotid artery imaging ≤2 d after presentation or in last 6 mo^b^	0.49 (0.38-0.63)	0.89 (0.73-1.09)	0.61 (0.52-0.72)	0.94 (0.80-1.10)
Carotid stenosis procedure ≤14 d among patients with procedure within 1 y	…^c^	0.18 (0.04-0.80)^d^	0.82 (0.16-4.15)	0.12 (0.03-0.49)^d^
Blood pressure				
Hypertension medication intensification ≤2 d after discharge^b^	0.55 (0.28-1.07)	0.97 (0.69-1.38)	0.82 (0.58-1.17)	1.04 (0.78-1.37)
Mean blood pressure of <140/90 mm Hg during 90 d postdischarge	NA	NA	1.04 (0.79-1.37)	0.69 (0.53-0.90)^d^
Guideline-concordant antihypertensive medication class ≤2 d after discharge^15^	0.58 (0.45-0.74)	0.74 (0.60-0.91)^d^	0.70 (0.60-0.83)	0.85 (0.72-0.99)^d^
Lipid management				
Lipid measurement ≤2 d after presentation or in prior 180 d	0.68 (0.51-0.90)	0.78 (0.62-0.99)^d^	0.64 (0.53-0.78)	0.84 (0.70-1.02)
Lipid management ≤2 d after discharge	0.46 (0.33-0.65)	0.73 (0.56-0.96)^d^	0.67 (0.53-0.85)	0.86 (0.68-1.07)
Discharged receiving statin ≤2 d after discharge	0.51 (0.36-0.73)	0.93 (0.70-1.23)	0.70 (0.55-0.88)	1.03 (0.81-1.30)
Cholesterol-lowering medication intensification ≤2 d after discharge	0.47 (0.26-0.83)	1.01 (0.75-1.36)	0.56 (0.41-0.77)	1.18 (0.93-1.48)
High- or moderate-potency statin ≤2 d after discharge^b^	0.83 (0.62-1.12)	0.73 (0.57-0.92)^d^	0.87 (0.72-1.05)	0.79 (0.66-0.96)^d^
Diabetes				
Hypoglycemic medication intensification ≤30 d after discharge	0.14 (0.01-5.49)	0.87 (0.26-2.87)	1.44 (0.64-3.23)	1.57 (0.81-3.05)
HbA_1c_ measurement by discharge or within prior 120 d among patients with diabetes	0.93 (0.60-1.44)	1.24 (0.86-1.80)	0.83 (0.63-1.10)	1.16 (0.88-1.55)
Cardiac monitoring				
Electrocardiography ≤2 d after presentation or ≤1 d before presentation	0.92 (0.70-1.21)	1.13 (0.91-1.40)	0.84 (0.71-1.00)^d^	1.12 (0.95-1.33)
Telemetry ≤2 d after presentation	1.02 (0.73-1.43)	1.22 (0.95-1.57)	1.01 (0.82-1.25)	1.04 (0.86-1.27)
Holter monitor recording ≤30 d after discharge	1.04 (0.37-2.89)	0.61 (0.23-1.67)	0.76 (0.44-1.30)	0.49 (0.26-0.92)^d^
Antithrombotics				
≤2 d after presentation	0.56 (0.40-0.79)	0.92 (0.69-1.21)	0.69 (0.55-0.87)	1.02 (0.81-1.28)
≤2 after discharge^b^	0.59 (0.41-0.86)	0.87 (0.65-1.16)	0.69 (0.54-0.88)	1.00 (0.79-1.28)
Anticoagulation				
Anticoagulation for atrial fibrillation ≤2 d after discharge^b^	0.59 (0.35-0.99)^d^	0.70 (0.39-1.26)	0.59 (0.40-0.85)	0.67 (0.42-1.08)
INR measurement ≤30 d after discharge	…^c^	0.25 (0.03-2.19)	1.41 (0.28-6.99)	1.06 (0.13-8.64)
Anticoagulation quality, INR 2-3 ≤30 after discharge	0.91 (0.26-3.24)	0.49 (0.09-2.66)	0.56 (0.32-1.00)	1.20 (0.51-2.78)
Brain imaging ≤2 d after presentation^b^	1.11 (0.75-1.63)	1.10 (0.83-1.47)	0.85 (0.67-1.07)	1.17 (0.93-1.49)
Deep vein thrombosis prophylaxis ≤2 d after admission among admitted patients	0.80 (0.55-1.15)	1.70 (1.17-2.49)^d^	0.84 (0.65-1.08)	1.21 (0.92-1.58)
Rehabilitation needs assessment ≤7 d after presentation	1.13 (0.84-1.53)	1.16 (0.89-1.50)	1.05 (0.88-1.26)	1.19 (0.99-1.43)
Speech-language pathology consultation before discharge among admitted patients	1.21 (0.89-1.65)	1.05 (0.82-1.34)	1.07 (0.87-1.30)	1.11 (0.91-1.34)
Substance use treatment referral for alcohol use before discharge	1.01 (0.13-8.02)	1.72 (0.64-4.64)	0.97 (0.28-3.36)	1.63 (0.72-3.69)
Nicotine replacement therapy ≤2 d after discharge among smokers	1.35 (0.82-2.21)	0.98 (0.70-1.36)	1.08 (0.78-1.48)	1.08 (0.82-1.40)
Polysomnography ≤90 d after presentation	NA	NA	0.05 (0-88.13)	1.09 (0.26-4.58)
Neurology consultation ≤1 d after presentation	0.67 (0.52-0.87)	0.92 (0.75-1.13)	0.74 (0.63-0.87)	0.98 (0.83-1.15)
Without-fail processes^b^	0.65 (0.45-0.94)^d^	0.74 (0.55-0.99)^d^	0.99 (0.55-0.87)	0.81 (0.65-1.01)

Abbreviations: HbA_1c_, hemoglobin A_1c_; INR, international normalized ratio; NA, not applicable; OR, odds ratio; ellipsis, not calculated.

^a^ Each risk-adjusted OR was generated from a single multivariable model that included all of the covariates listed in Table 2.

^b^ Overall, 6 processes of care were included in the without-fail measure: carotid artery imaging, hypertension medication intensification, high- or moderate-potency statin therapy, brain imaging, antithrombotics at discharge, and anticoagulation for atrial fibrillation. A patient passed the without-fail rate if they received all of the processes for which they were eligible.

^c^ Too few patients were included to calculate 90-day mortality.

^d^ *P *value for this process of care and outcome was not statistically significant following false discovery rate adjustment for multiple comparisons. All raw and adjusted *P* values for each comparison are provided in eTable 8 in the Supplement.

### Without-Fail Care

Among 8076 patients, 143 were not eligible for at least 1 of the 6 without-fail processes, leaving 7933 eligible. The without-fail rate was 15.3%, meaning that only 1216 patients (15.3%) received all the processes of care for which they were eligible among the 6 without-fail processes. Receiving without-fail care was associated with a 31.2% reduction in the odds of 1-year mortality (aOR, 0.69; 95% CI, 0.55-0.87) but was not independently associated with 90-day mortality or stroke outcomes after adjustment for multiple comparisons (Table 3) (eTable 8 in the Supplement).

### Sensitivity Analysis

In analyses restricted to patients 65 years and older, the results were similar to the main results (eTable 6 in the Supplement). The magnitude of the association of the without-fail care rate with outcomes was similar to the main analysis, but the aORs were modestly higher when restricted to the population 65 years and older (eTable 6 in the Supplement). The magnitude of the association of the without-fail care rate with mortality was nearly identical when comparing patients with TIA with patients with nonsevere ischemic stroke; however, the aORs for the without-fail care rate and recurrent stroke risk were somewhat lower among patients with nonsevere ischemic stroke compared with patients with TIA (eTable 6 in the Supplement). For medication-based processes, the aORs were very similar for patients who were taking medication before the index event and patients who were given a new prescription after the index event (eTable 7 in the Supplement).

## Discussion

These results support the association of guideline-concordant processes with improved outcomes for patients with TIA and nonsevere ischemic stroke. The 6 without-fail care processes (ie, brain imaging, carotid artery imaging, antihypertensive intensification, high- or moderate-potency statin therapy, antithrombotics, and anticoagulation for atrial fibrillation) can be provided routinely at diverse medical centers because they do not require specialized structures of care. Given the strength of the prospective trial evidence as well as the current findings supporting the association of these processes with improved outcomes, health care systems should prioritize providing patients with TIA or nonsevere ischemic stroke with the guideline-concordant processes of care for which they are eligible.

The individual processes that were associated with improved outcomes included carotid stenosis management, hyperlipidemia, anticoagulation for atrial fibrillation, antithrombotics, and neurology consultation. These data support the AHA/ASA secondary prevention recommendations.^8^ Although carotid artery screening and intervention for symptomatic carotid stenosis are endorsed by guidelines, these processes are not currently the focus of existing quality measurement programs.^8,13^

The observed recurrent event rates are similar to those from other settings.^4,6,7,21^ For example, 90-day recurrent TIA and stroke rates have been reported in the 8.5%^4^ to 9.9%^7^ range. Johnston et al^21^ reported a 90-day stroke rate of 10.5% for patients with TIA, but those data were from a cohort in 1997 to 1998. However, data from Australia indicated that only 4.2% of patients with TIA or nonsevere ischemic stroke who were treated in stroke units had recurrent stroke in 90 days;^22^ it may be that high-quality care provided in specialized stroke units conferred a lower stroke recurrence rate.^23,24^ The TIAregistry.org project (2009-2011) reported a 90-day recurrent stroke rate of 3.7%, a 1-year recurrent stroke rate of 5.1%, and a 1-year mortality rate of 1.8%; most patients (78%) received early stroke specialist care.^25^ We similarly observed that early neurology consultation was associated with a reduction in mortality.^23^ Therefore, the difference in risk of mortality between the TIAregistry.org project and the current cohort may be owing to differences in neurology consultation rates. We relied on emergency department and inpatient use to identify recurrent stroke events; therefore, the reported recurrent stroke rates underestimate actual rates for patients seeking care at non-VHA facilities. It was for this reason that we conducted sensitivity analyses focused on patients 65 years and older for whom we had additional health care utilization data; the results of this analysis supported the overall findings.

We were surprised that not more individual processes of care were independently associated with improved outcomes. Our analyses may have been limited by relatively high pass rates on several processes. For example, although antithrombotic medications were associated with lower odds of mortality, they were not associated with recurrent stroke risk. A meta-regression of trials from the 1970s to 1990s^26^ indicated that approximately 14% of patients with stroke have a recurrent stroke during 32 months of follow-up, and the recurrent stroke risk can be reduced by 15% (95% CI, 6%-23%) with aspirin. It may be that our cohort of patients with TIA and nonsevere ischemic stroke, who had a 1-year recurrent stroke rate of 10.7% and a pass rate of antithrombotics at discharge of 87%, may have included too few eligible patients who did not receive antithrombotics to detect differences in recurrent stroke risk. However, among 67 892 patients in the Get With the Guidelines–Stroke cohort, aspirin use was also not statistically associated with a reduction in 1-year recurrent stroke risk.^18^ Therefore, it may be that the effect of antithrombotics on stroke risk is less robust now than was observed in the clinical trials, many of which were conducted before the advent of high-potency statins.

Although several risk prediction scores have been validated for patients with stroke and TIA, we did not rely on any single risk adjustment score.^1,3,22,27,28,29^ Because the clinical features of cerebrovascular events are not routinely captured in electronic health record data, we could not use the age, blood pressure, clinical features, duration of symptoms, and diabetes (ABCD^2^) score, but we did include 3 of its components (age, blood pressure, diabetes). We evaluated a comprehensive set of risk-adjustment variables taken from the literature about post-TIA risk prediction. The final model included variables with robust associations with postevent outcomes (Table 2).

Given that these data were derived from an observational cohort, we cannot exclude the possibility of confounding. We must consider that unmeasured confounders bias the assessment of the associations of patient characteristics with outcomes as well as of processes with outcomes. The observation that some clinical characteristics were associated with lower odds of death might be because of confounding. For example, patients with prior carotid endarterectomy or carotid stent had lower odds of 90-day mortality than patients without carotid interventions (Table 2). It may be that surgeons favor patients with strong performance status, and thereby, the carotid intervention variable is a surrogate for good health. Although we included a broad range of patient characteristics in the risk adjustment, we recognize that the risk of confounding cannot be eliminated in observational cohort studies.

A related methodological issue is confounding by indication for the processes. We observed that patients who were not eligible for a given process generally had higher odds of poor outcomes than patients who were eligible for that process. For example, patients who were eligible for carotid imaging and received the process had lower odds of 90-day mortality compared with patients who were eligible but did not receive carotid imaging (aOR, 0.49; 95% CI, 0.38-0.63). However, patients who were not eligible for carotid imaging had higher odds of 90-day mortality compared with patients who were eligible but did not receive the process (eTable 2 in the Supplement). This observation is clinically expected because patients with very high disease severity (eg, hospice) were generally not eligible for processes (eTable 1 in the Supplement). Similar to findings from other cohort studies, we also observed that some processes were associated with an increased risk of adverse events.^30^ For example, the observation that rehabilitation needs assessment was associated with a higher odds of recurrent stroke (albeit not statistically significant) may be owing to confounding by stroke severity (ie, patients with severe deficits were more likely to have rehabilitation consultations); we did not have a measure of disease severity in the risk-adjustment model.

We could have used 2 alternative methodological strategies: (1) the cohort could have been restricted to patients who were eligible for all processes of care, or (2) propensity analyses could have been conducted for each process. We favored including all patients in the study because some key processes (eg, anticoagulation for atrial fibrillation) are relevant to small subpopulations (ie, patients with atrial fibrillation), limiting the sample size and the generalizability. Propensity score adjustment is used to reduce bias when estimating the effect of an intervention on an outcome and is generally applied when investigating associations of single interventions with outcomes, especially when the characteristics of patients who receive an intervention differ from patients who do not receive an intervention.^31^ Given that we were interested in evaluating a broad range of processes, propensity score adjustment would have necessitated the development and presentation of more than 2 dozen models. Our approach involved defining eligibility for processes based on clinical guidelines. The risk-adjusted results (Table 3) describe the association of processes of care with outcomes among patients who were eligible for those processes.

### Strengths and Limitations

The strengths of this study were its national scope, relatively large sample size, and inclusion of a comprehensive set of processes as well as a bundle of care. This study had limitations. First, as described earlier, although the modeling included a broad range of potential confounders,^32^ the possibility for residual confounding exists, especially because we did not have brain imaging results^33^ or data on stroke severity, stroke type, or stroke location. Second, without-fail processes were identified a priori; therefore, it is possible that the without-fail measure did not include processes with the strongest associations with outcomes. Future studies might consider identifying all-or-none quality measures empirically. Third, this study focused on veterans seeking care within VHA facilities, so the results may not generalize to other populations or health care settings.^34^

## Conclusions

In this study, individual processes of care and a 6-item without-fail care measure were associated with clinically meaningful reductions in risk of stroke and death. Widespread implementation of these processes should be strongly considered for patients with TIA and nonsevere ischemic stroke. In addition, health care systems should consider routinely measuring key processes of care for patients with TIA in addition to the quality measurement that exists for patients with stroke.

## Notes

Supplement. **eTable 1. **Process of Care Numerator, Denominator, and Exclusions Definitions **eTable 2. **Multivariable Modeling Results Including Patients Ineligible for Processes of Care: 90-Day Mortality **eTable 3. **Multivariable Modeling Results Including Patients Ineligible for Processes of Care: 1-Year Mortality **eTable 4. **Multivariable Modeling Results Including Patients Ineligible for Processes of Care: 90-Day Recurrent Stroke **eTable 5.** Multivariable Modeling Results Including Patients Ineligible for Processes of Care: 1-Year Recurrent Stroke **eTable 6. **Sensitivity Analyses: Adjusted Association of Without-Fail Rate With Outcomes **eTable 7. **Sensitivity Analyses: Adjusted Analyses Between Medication Categories and Outcomes **eTable 8.** Adjusted P Values Based on the False Discovery Rate
