# Supplementary material for: Processes of Care Associated With Risk of Mortality and Recurrent Stroke Among Patients With Transient Ischemic Attack and Nonsevere Ischemic Stroke
Source: JAMA Netw Open. 2019 Jul 3;2(7):e196716. doi: 10.1001/jamanetworkopen.2019.6716 (PMC6613337; doi:10.1001/jamanetworkopen.2019.6716)
Supplement: Supplement. — eTable 1. Process of Care Numerator, Denominator, and Exclusions Definitions eTable 2. Multivariable Modeling Results Including Patients Ineligible for Processes of Care: 90-Day Mortality eTable 3. Multivariable Modeling Results Including Patients Ineligible for Processes of Care: 1-Year Mortality eTable 4. Multivariable Modeling Results Including Patients Ineligible for Processes of Care: 90-Day Recurrent Stroke eTable 5. Multivariable Modeling Results Including Patients Ineligible for Processes of Care: 1-Year Recurrent Stroke eTable 6. Sensitivity Analyses: Adjusted Association of Without-Fail Rate With Outcomes eTable 7. Sensitivity Analyses: Adjusted Analyses Between Medication Categories and Outcomes eTable 8. Adjusted P Values Based on the False Discovery Rate [file jamanetwopen-2-e196716-s001.pdf]

Supplementary Online Content

Bravata DM, Myers LJ, Reeves M, et al. Processes of care associated with risk of mortality and recurrent stroke among patients with transient ischemic attack and nonsevere ischemic stroke. *JAMA Netw Open*. 2019;2(7):e196716. doi:10.1001/jamanetworkopen.2019.6716

eTable 1. Process of Care Numerator, Denominator, and Exclusions Definitions

eTable 2. Multivariable Modeling Results Including Patients Ineligible for Processes of Care: 90-Day Mortality

eTable 3. Multivariable Modeling Results Including Patients Ineligible for Processes of Care: 1-Year Mortality

eTable 4. Multivariable Modeling Results Including Patients Ineligible for Processes of Care: 90-Day Recurrent Stroke

eTable 5. Multivariable Modeling Results Including Patients Ineligible for Processes of Care: 1-Year Recurrent Stroke

eTable 6. Sensitivity Analyses: Adjusted Association of Without-Fail Rate With Outcomes

eTable 7. Sensitivity Analyses: Adjusted Analyses Between Medication Categories and Outcomes

eTable 8. Adjusted P Values Based on the False Discovery Rate

This supplementary material has been provided by the authors to give readers additional information about their work.

© 2019 Bravata DM et al. *JAMA Network Open.*

eTable 1. Process of Care Numerator, Denominator, and Exclusions Definitions

Label	Numerator	Denominator	Exclusions
Carotid Artery Imaging	Patients receiving a carotid imaging procedure within 2 days after presentation or 6 months before presentation	Transient ischemic attack (TIA) or non-severe stroke patient	• Died within 2 days of presentation<br>• Transferred to a facility not within the Veterans Health Administration (Non-VA) acute care facility within 2 days of presentation<br>• Discharged to hospice<br>• Patients who left against medical advice within 2 days of presentation
Carotid Stenosis Procedure	Patients who receive a carotid stenosis procedure (endarterectomy or stent) within 14 days of presentation	TIA or non-severe stroke patient who received carotid endarterectomy or carotid stent within 365 days of presentation	• Died during index TIA/non-severe stroke hospitalization or within 14 days of presentation<br>• Discharged to hospice<br>• Transferred to another Non-VA acute care facility within 14 days of presentation<br>• Patients who left against medical advice (AMA) within 14 days of presentation
Hypertension Medication Intensification	Patients who received intensification of antihypertensive medications (new drug or higher dosage of existing drug with a supply >1day) within 2 days of discharge<br><br>(A 90-day look-back period from time of index event is used to compare filled medications 90 days pre-event with 90-days post-event. If the same medications/dosage were prescribed, these medications should be considered present at the time of the index event. If the medication supply does not extend to the time of admission, the medication is not considered present upon admission.)	TIA or non-severe stroke patient with blood pressure above goal (systolic blood pressure ≥140 **OR** diastolic blood pressure ≥90 mm Hg) at the time of discharge	• Died during index TIA/non-severe stroke hospitalization or within 7 days of discharge<br>• Discharged to hospice<br>• Transferred to another Non-VA acute care facility<br>• Patients on dialysis 6 months pre-event, during the hospital stay or within 6 months after presentation<br>• Missing pre-visit medication data<br>• Patients with missing post-discharge medication data<br>• Patients already on 4 antihypertensive medications<br>• Patient without any valid blood pressure measurements<br>• Patients who left against medical advice
Hypertension Control	Patients whose calculated mean blood pressure from the values in the 90-days post-discharge is systolic blood pressure <140 mmHg **AND** diastolic blood pressure <90 mmHg	TIA or non-severe stroke patient	• Died during index TIA/non-severe stroke hospitalization or within 90 days of discharge<br>• Discharged to hospice<br>• Transferred to another Non-VA acute care facility
Antihypertensive Medication Class at Discharge[33]	Appropriate antihypertensive medication class was prescribed at discharge or within 2 days of discharge	TIA or non-severe stroke patient with a diagnosis of hypertension within 2 years of index event (≥1 ICD9 code in the inpatient setting, or ≥2 codes in the outpatient setting over ≥30-day period)	• Transferred to another Non-VA acute care facility<br>• Patients with a missing or unknown race

© 2019 Bravata DM et al. *JAMA Network Open.*

eTable 1. Process of Care Numerator, Denominator, and Exclusions Definitions (continued)

Label	Numerator	Denominator	Exclusions
Lipid Measurement	Patients who have their lipids measured within 2 days of presentation unless there is a measurement available from within the prior 180 days	TIA or non-severe stroke patient	• Died within 2 days of presentation<br>• Discharged to hospice<br>• Transferred to another Non-VA acute care facility within 2 days of presentation<br>• Patients who left against medical advice within 2 days of presentation
Lipid Management	Patients who were prescribed cholesterol-reducing therapy (with an outpatient supply >1 day) within 2 days after discharge<br><br>(A 90-day look-back period from the time of the index event is used in order to compare filled medications 90-days pre-event with 90-days post-event. If the same medications/dosage were prescribed, these medications should be considered present at the time of the index event. If the medication supply does not extend to the time of admission, the medication is not considered present upon admission)	TIA or non-severe stroke patient with inpatient LDL-cholesterol ≥100mg/dL **OR IF** inpatient LDL-cholesterol is <100mg/dL or missing patients who were on cholesterol-reducing therapy prior to index event **OR IF** inpatient LDL-cholesterol is missing and patient was not on cholesterol-reducing therapy prior to index event, patients who had LDL-cholesterol ≥100mg/dL within 180 days prior to index event **OR** LDL not measured in prior 180 days	• Transferred to another Non-VA acute care facility<br>• Patients who died during the hospital stay or Emergency Department (ED) visit<br>• Patients who left against medical advice<br>• Patients discharged to hospice<br>• Patients with allergy to statin therapy
Discharged on Statin	Patients who were prescribed a statin medication (with an outpatient supply >1 day) within 2 days after discharge<br><br>(A 90-day look-back period from the time of the index event is used in order to compare filled medications 90-days pre-event with 90-days post-event. If the same medications/dosage were prescribed, these medications should be considered present at the time of the index event. If the medication supply does not extend to the time of admission, the medication is not considered present upon admission)	TIA or non-severe stroke patient with inpatient LDL-cholesterol ≥100mg/dL **OR IF** inpatient LDL-cholesterol is <100mg/dL or missing patients who were on cholesterol-reducing therapy prior to index event **OR IF** inpatient LDL-cholesterol is missing and patient was not on cholesterol-reducing therapy prior to index event, patients who had LDL-cholesterol ≥100mg/dL within 180 days prior to index event **OR** LDL-cholesterol not measured in prior 180 days	• Transferred to another Non-VA acute care facility<br>• Patients who died during the hospital stay or ED visit<br>• Patients who left against medical advice<br>• Patients discharged to hospice<br>• Patients with allergy to statin therapy

© 2019 Bravata DM et al. *JAMA Network Open.*

eTable 1. Process of Care Numerator, Denominator, and Exclusions Definitions (continued)

Label	Numerator	Denominator	Exclusions
Cholesterol Lowering Intensification	Patients who received intensification of cholesterol-reducing therapy within 2 days after discharge defined as either addition of a new cholesterol-reducing medication or increased dose of prior medication<br><br>(A 90-day look-back period from the time of the index event is used in order to compare filled medications 90-days pre-event with 90-days post-event. If the same medications/dosage were prescribed, these medications should be considered present at the time of the index event. If the medication supply does not extend to the time of admission, the medication is not considered present upon admission.)	TIA or non-severe stroke patient, **AND**<br><br>Patients with LDL-cholesterol ≥100mg/dL during hospital (index date or 1 day after for ED patients), **OR**<br><br>Patients who were on cholesterol-reducing therapy at time of the index event	• Transferred to another Non-VA acute care facility<br>• Patients who died during the hospital stay or during ED visit<br>• Patients who left against medical advice<br>• Patients discharged to hospice<br>• Patients with allergy to statin therapy<br>• Patients on atorvastatin 80mg<br>• Patients on rosuvastatin 40mg
High or Moderate Potency Statin at Discharge	Patients who receive statin therapy within 2 days after discharge (with >1 day of supply) defined as follows:<br><br>≤75 years of age: high potency statin<br><br>>75 years of age: moderate or high potency statin	TIA or non-severe stroke patient	• Transferred to another Non-VA acute care facility<br>• Patients who died during the hospital stay or during ED visit<br>• Patients who left against medical advice<br>• Patients discharged to hospice<br>• Patients with allergy to statin therapy
Brain Imaging	Patients receiving brain imaging (Brain CT or MRI) within 2 days of presentation	TIA or non-severe stroke patient	• Died within two days of presentation<br>• Discharged to hospice within 2 days of presentation<br>• Left AMA within 2 days of index event<br>• Transferred to another Non-VA acute care facility within 2 days of presentation<br>• Admitted from a Non-VA acute care facility
Electrocardiography	Patients receiving an electrocardiogram one day before index event or within 2 days of presentation	TIA or non-severe stroke patient	• Died within two days of index event<br>• Discharged to hospice within 2 days of presentation<br>• Left AMA within 2 days of presentation<br>• Transferred to another Non-VA acute care facility within 2 days of presentation<br>• Admitted from a Non-VA acute care facility

© 2019 Bravata DM et al. *JAMA Network Open.*

eTable 1. Process of Care Numerator, Denominator, and Exclusions Definitions (continued)

Label	Numerator	Denominator	Exclusions
Telemetry	Patients receiving telemetry within 2 days of presentation or admitted to intensive care unit during index hospitalization	TIA or non-severe stroke patient who was admitted to the hospital	• Died within two days of presentation<br>• Discharged without being admitted<br>• Discharged to hospice<br>• Transferred to Non-VA acute care facility within 2 days of presentation<br>• Admitted from a Non-VA acute care facility<br>• Patients who left AMA within 2 days of presentation<br>• Patients with atrial fibrillation (At least 1 inpatient code or at least 2 outpatient codes in the prior 5 years)
Holter	Patients who receive a Holter monitor or cardiac event monitor within 30 days of discharge	TIA or non-severe stroke patient	• Died within 30 days of discharge<br>• Discharged to hospice<br>• Transferred to a Non-VA acute care facility<br>• Patients with atrial fibrillation (At least 1 inpatient code or at least 2 outpatient codes in the prior 5 years)
Antithrombotics by Day 2	Patients who receive antithrombotic therapy by the end of hospital day two or within 2 days of presentation	TIA or non-severe stroke patient	• Died with first two days of presentation<br>• Discharged to hospice<br>• Transferred to another Non-VA acute care facility within 2 days of presentation<br>• Patients who left AMA within 2 days of presentation<br>• Patients with an allergy to antithrombotic medication<br>• Patients who receive tPA during index stay
Antithrombotics at Discharge	Patients who receive antithrombotic therapy within 2 days after discharge (with an outpatient supply >1 day)	TIA or non-severe stroke patient	• Died during hospital stay<br>• Discharged to hospice<br>• Transferred to another Non-VA acute care facility<br>• Patients who left against medical advice<br>• Patients with an allergy to antithrombotic medication<br>• Patients who received thrombolysis within 2 days of discharge
Anticoagulation for Atrial Fibrillation/Flutter	Patients prescribed anticoagulation therapy within 2 days after discharge	• TIA or non-severe stroke patient **AND** one or more of the following:<br>- Atrial fibrillation/paroxysmal atrial fibrillation during hospital stay<br>- Discharge diagnosis of atrial fibrillation<br>- History of atrial fibrillation/paroxysmal atrial fibrillation prior to hospital stay (Must have at least 1 inpatient code or at least 2 outpatient codes in the prior 5 years)	• Transferred to another Non-VA acute care facility<br>• Patients who died during hospital stay or ED visit<br>• Patients who left against medical advice<br>• Patients discharged to hospice<br>• Patients with an allergy to any anticoagulant<br>• Patients who receive thrombolysis within 2 days of discharge<br>• Patients on dialysis 6 months pre-event, during the hospital stay or within 6 months after the event

© 2019 Bravata DM et al. *JAMA Network Open.*

eTable 1. Process of Care Numerator, Denominator, and Exclusions Definitions (continued)

Label	Numerator	Denominator	Exclusions
INR Measured for patients with atrial fibrillation on warfarin	INR measured within 30 days of discharge	• TIA or non-severe stroke patient **AND**<br>• Atrial fibrillation **AND**<br>- Atrial fibrillation/paroxysmal atrial fibrillation during hospital stay<br>- Discharge diagnosis of atrial fibrillation<br>- History of atrial fibrillation/paroxysmal atrial fibrillation prior to hospital stay (Must have at least 1 inpatient code or at least 2 outpatient codes in the prior 5 years)<br>• On warfarin at discharge or within 30 days of discharge	• Transferred to a Non-VA acute care facility<br>• Patients who died during hospital stay or ED visit or within 30 days of discharge<br>• Patients discharged to hospice
Anticoagulation Quality	INR ≥2.0 and ≤3.0 within 30 days of discharge<br><br>(the last INR measurement within 30 days of discharge is used)	• TIA or non-severe stroke patient **AND**<br>• Atrial fibrillation **AND**<br>- Atrial fibrillation/paroxysmal atrial fibrillation during hospital stay<br>- Discharge diagnosis of atrial fibrillation<br>- History of atrial fibrillation/paroxysmal atrial fibrillation prior to hospital stay (Must have at least 1 inpatient code or at least 2 outpatient codes in the prior 5 years)<br>• On warfarin at discharge or within 30 days of discharge<br>• International Normalized Ratio (INR) measured 30 days of discharge	• Transferred to another Non-VA acute care facility<br>• Patients who died during hospital stay or ED visit or within 30 days of discharge<br>• Patients discharged to hospice
HBA1c Measurement	Patients who have their HbA1c (glycosylated hemoglobin) measured within 2 days of presentation (if a measurement is not available from the prior 120 days)<br><br>(If there is more than one HbA1c measured, use the last value)	• TIA or minor stroke patient **AND**<br>• Patients with diabetes mellitus identified either via history within 2 years (≥1 ICD9 code in the inpatient setting, or ≥2 codes in the outpatient setting over ≥30 day period) or use of insulin or oral hypoglycemic medications within 1 year of the index event (presentation or discharge)	• Died within 2 days of presentation<br>• Discharged to hospice<br>• Transferred to another Non-VA acute care facility within 2 days of presentation<br>• Patients who left against medical advice within 2 days of presentation

© 2019 Bravata DM et al. *JAMA Network Open.*

eTable 1. Process of Care Numerator, Denominator, and Exclusions Definitions (continued)

Label	Numerator	Denominator	Exclusions
Oral Hypoglycemic Medication Intensification	Patients who receive intensification of medication therapy (either new medication or increase in dose of prior medication with a supply >1day) within 30 days of discharge where a new Insulin prescription is considered as intensification<br><br>(A 90-day look-back period from the time of the index event is used in order to compare filled medications 90-days pre-event with 90-days post-event. If the same medications/dosage were prescribed, these medications should be considered present at the time of the index event. If the medication supply does not extend to the time of admission, the medication is not considered present upon admission.)	• TIA or minor stroke patient **AND**<br>• Patients with diabetes mellitus identified either via history within 2 years (≥1 ICD9 code in the inpatient setting, or ≥2 codes in the outpatient setting over ≥30-day period) or use of oral hypoglycemic medications within 1 year of the index event (presentation or discharge)<br>• HbA1c >7% measured at the time of the event (index date or 1 day after for ED patients) OR within 120 days before the event<br>*If there is more than one HbA1c measured, take the last value	• Transferred to another Non-VA acute care facility<br>• Patients who died during the hospital stay or during ED visit<br>• Patients who left against medical advice<br>• Patients discharged to hospice<br>• Patients on Insulin at the time of the index event (within 120 days pre-event)
Deep Vein Thrombosis (DVT) Prophylaxis	Patients who had DVT prophylaxis initiated by end of hospital day 2	TIA or non-severe stroke patient	• Patients who were not admitted (ED visit only)<br>• Patients discharged prior to end of hospital day 2<br>• Patients discharged to hospice<br>• Transferred to another Non-VA acute care facility within 2 days of index event<br>• Patients who receive thrombolysis during index stay
Speech-Language Pathology Consultation	Receipt of speech-language pathology consultation during inpatient period	TIA or non-severe stroke patient	• Patients who were not admitted (ED visit only)<br>• Patients who died during hospital stay<br>• Patient discharged to hospice<br>• Transferred to another Non-VA acute care facility<br>• Patients left AMA
Substance Use Treatment Referral for Alcohol Use	Order for substance use treatment clinic consultation prior to discharge	• TIA or non-severe stroke patient **AND**<br>• Alcohol dependence or alcohol abuse within 6 months of index event	• Patients who died during hospital stay<br>• Patient discharged to hospice<br>• Transferred to another Non-VA acute care facility<br>• Patients left AMA
Nicotine Replacement Therapy	Patients prescribed nicotine replacement therapy (Nicotine or Varenicline) or received a smoking cessation clinic consult within 2 days after discharge	• TIA or non-severe stroke patient **AND**<br>• Tobacco use (look back period of 1-year)	• Transferred to another Non-VA acute care facility<br>• Patients who died during hospital stay or ED visit<br>• Patients who left against medical advice<br>• Patients discharged to hospice<br>• Patients who refuse smoking cessation

© 2019 Bravata DM et al. *JAMA Network Open.*

eTable 1. Process of Care Numerator, Denominator, and Exclusions Definitions (continued)

Label	Numerator	Denominator	Exclusions
Polysomnography	Polysomnography receipt within 90 days of presentation	TIA or non-severe stroke patient	• Patients who died during hospital stay or ED visit<br>• Patients discharged to hospice<br>• Transferred to another Non-VA acute care facility<br>• Patients who left against medical advice<br>• Patients with history of sleep apnea within 5 years of index event<br>• Patients with prior polysomnography within 5 years of index event
Neurology Consultation	Patients who receive a neurology consultation within one day after presentation	TIA or non-severe stroke patient	• Transferred to another Non-VA acute care facility within 2 days of presentation
Rehabilitation Needs Assessment	Receipt of rehabilitation consultation (physical therapy, occupational therapy, speech therapy, kinesiotherapy, physical medicine & rehabilitation consult) during inpatient stay or within 7 days of presentation	TIA or non-severe stroke patient	• Died within 7 days of presentation<br>• Transferred to another Non-VA acute care facility within 7 days of presentation Discharged to hospice<br>• Patients who left against medical advice within 7 days of presentation

© 2019 Bravata DM et al. *JAMA Network Open.*

eTable 2. Multivariable Modeling Results Including Patients Ineligible for Processes of Care: 90-Day Mortality

					Univariate Models				Multivariable			
Process	Effect	Pass	Eligible	Pass Rate	Estimate	Std Error	p-value	Odds Ratio (95% Confidence Interval)	Estimate	Std Error	p-value	Odds Ratio (95% Confidence Interval)
Carotid Artery Imaging	Pass	4667	7932	0.5884	-0.763	0.122	<0.001	0.466 (0.367, 0.592)	-0.716	0.130	<0.001	0.489 (0.379, 0.631)
	Not eligible				1.322	0.237	<0.001	3.749 (2.358, 5.962)	1.507	0.268	<0.001	4.512 (2.672, 7.621)
Carotid Stenosis Procedure	Pass	63	279	0.2258	-	-	-	-	-	-	-	-
	Not eligible				0.380	0.511	0.458	1.463 (0.538, 3.974)	-0.035	0.524	0.947	0.966 (0.346, 2.691)
Hypertension Management	Pass	664	2823	0.2352	-0.623	0.330	0.060	0.536 (0.282, 1.022)	-0.597	0.340	0.081	0.551 (0.283, 1.071)
	Not eligible				0.426	0.142	0.003	1.531 (1.160, 2.020)	0.425	0.149	0.005	1.530 (1.143, 2.049)
Hypertension Control	Pass	4666	6139	0.7601	-	-	-	-	-	-	-	-
	Not eligible				-	-	-	-	-	-	-	-
Antihypertensive Medication Class	Pass	3894	7186	0.5419	-0.659	0.122	<0.001	0.517 (0.408, 0.656)	-0.551	0.130	<0.001	0.576 (0.447, 0.743)
	Not eligible				-0.954	0.239	<0.001	0.385 (0.241, 0.615)	-0.182	0.275	0.510	0.834 (0.487, 1.428)
Lipid Measurement	Pass	6334	7933	0.7984	-0.409	0.135	0.003	0.664 (0.510, 0.864)	-0.389	0.145	0.008	0.678 (0.510, 0.900)
	Not eligible				1.401	0.252	<0.001	4.059 (2.481, 6.642)	1.588	0.284	<0.001	4.894 (2.808, 8.529)
Lipid Management	Pass	5001	6174	0.8100	-0.572	0.159	<0.001	0.564 (0.413, 0.770)	-0.772	0.174	<0.001	0.462 (0.329, 0.650)
	Not eligible				0.217	0.166	0.192	1.242 (0.898, 1.718)	-0.039	0.180	0.829	0.962 (0.677, 1.367)
Discharged on Statin	Pass	4992	6174	0.8086	-0.487	0.161	0.003	0.615 (0.448, 0.843)	-0.665	0.176	<0.001	0.514 (0.364, 0.726)
	Not eligible				0.280	0.168	0.098	1.323 (0.952, 1.839)	0.040	0.182	0.826	1.041 (0.729, 1.486)
Cholesterol Lowering Intensification	Pass	1146	4643	0.2468	-1.083	0.285	<0.001	0.338 (0.194, 0.591)	-0.764	0.293	0.010	0.466 (0.263, 0.826)
	Not eligible				0.435	0.120	<0.001	1.544 (1.222, 1.952)	0.543	0.132	<0.001	1.720 (1.330, 2.225)
High or Moderate Potency Statin at Discharge	Pass	2251	7279	0.3093	0.295	0.134	0.029	1.343 (1.032, 1.746)	-0.181	0.149	0.224	0.834 (0.624, 1.116)
	Not eligible				1.105	0.151	<0.001	3.020 (2.249, 4.054)	0.958	0.168	<0.001	2.606 (1.874, 3.623)
Brain Imaging	Pass	6795	7849	0.8657	0.032	0.179	0.857	1.033 (0.728, 1.465)	0.102	0.197	0.608	1.107 (0.753, 1.628)
	Not eligible				1.491	0.259	<0.001	4.443 (2.680, 7.365)	1.504	0.288	<0.001	4.501 (2.563, 7.903)
Electrocardiography	Pass	5196	7851	0.6618	-0.012	0.132	0.926	0.988 (0.764, 1.278)	-0.086	0.140	0.539	0.918 (0.698, 1.206)
	Not eligible				1.465	0.225	<0.001	4.329 (2.787, 6.723)	1.376	0.251	<0.001	3.959 (2.423, 6.469)
Telemetry	Pass	2417	4932	0.4901	-0.027	0.167	0.871	0.973 (0.702, 1.349)	0.022	0.172	0.899	1.022 (0.730, 1.432)
	Not eligible				0.430	0.140	0.002	1.537 (1.169, 2.020)	0.161	0.172	0.350	1.175 (0.839, 1.643)
Holter	Pass	341	6707	0.0508	-0.238	0.517	0.646	0.788 (0.287, 2.168)	0.035	0.524	0.947	1.036 (0.371, 2.889)
	Not eligible				1.125	0.196	<0.001	3.080 (2.100, 4.517)	-0.105	0.392	0.788	0.900 (0.418, 1.936)

© 2019 Bravata DM et al. *JAMA Network Open.*

eTable 2. Multivariable Modeling Results Including Patients Ineligible for Processes of Care: 90-Day Mortality (continued)

					Univariate Models				Multivariable			
Process	Effect	Pass	Eligible	Pass Rate	Estimate	Std Error	p-value	Odds Ratio (95% Confidence Interval)	Estimate	Std Error	p-value	Odds Ratio (95% Confidence Interval)
Antithrombotics by Hospital Day 2	Pass	6539	7645	0.8553	-0.310	0.158	0.051	0.734 (0.538, 1.000)	-0.574	0.172	0.001	0.563 (0.402, 0.788)
	Not eligible				0.692	0.222	0.002	1.998 (1.295, 3.082)	0.384	0.242	0.114	1.468 (0.914, 2.358)
Antithrombotics at Discharge	Pass	6584	7599	0.8664	-0.239	0.173	0.169	0.788 (0.562, 1.105)	-0.522	0.187	0.006	0.593 (0.412, 0.856)
	Not eligible				1.220	0.211	<0.001	3.385 (2.242, 5.112)	0.932	0.232	<0.001	2.539 (1.614, 3.995)
Anticoagulation for Atrial Fibrillation	Pass	829	1071	0.7740	-0.366	0.247	0.140	0.693 (0.428, 1.124)	-0.523	0.264	0.049	0.593 (0.353, 0.994)
	Not eligible				-1.259	0.220	<0.001	0.284 (0.185, 0.437)	-0.183	0.298	0.539	0.833 (0.465, 1.491)
INR Measured	Pass	466	486	0.9589	-	-	-	-	-	-	-	-
	Not eligible				-	-	-	-	-	-	-	-
Anticoagulation Quality	Pass	239	466	0.5129	-0.060	0.642	0.926	0.942 (0.268, 3.305)	-0.097	0.650	0.882	0.908 (0.255, 3.239)
	Not eligible				-0.245	0.463	0.597	0.783 (0.317, 1.935)	0.776	0.491	0.116	2.172 (0.831, 5.679)
HbA1c Measurement	Pass	2593	3297	0.7865	-0.178	0.209	0.395	0.837 (0.556, 1.260)	-0.071	0.223	0.749	0.931 (0.602, 1.439)
	Not eligible				-0.145	0.197	0.462	0.865 (0.589, 1.271)	0.450	0.276	0.104	1.569 (0.914, 2.691)
Hypoglycemic Medication	Pass	195	571	0.3415	-	-	-	-	-	-	-	-
	Not eligible				0.325	0.460	0.481	1.384 (0.563, 3.401)	0.153	0.483	0.751	1.166 (0.453, 3.000)
Deep Vein Thrombosis (DVT) Prophylaxis	Pass	3451	4294	0.8037	-0.091	0.178	0.607	0.913 (0.645, 1.292)	-0.229	0.187	0.222	0.795 (0.551, 1.147)
	Not eligible				-0.551	0.184	0.003	0.577 (0.402, 0.827)	-0.286	0.196	0.147	0.751 (0.512, 1.103)
Rehabilitation Needs Assessment	Pass	3744	7671	0.4881	0.537	0.141	<0.001	1.711 (1.299, 2.255)	0.123	0.155	0.426	1.131 (0.836, 1.531)
	Not eligible				1.119	0.363	0.002	3.063 (1.505, 6.231)	1.198	0.389	0.002	3.314 (1.549, 7.093)
Speech-Language Pathology Consultation	Pass	2068	5653	0.3658	0.633	0.143	<0.001	1.884 (1.423, 2.493)	0.192	0.158	0.225	1.212 (0.890, 1.651)
	Not eligible				0.495	0.141	0.001	1.641 (1.246, 2.162)	0.656	0.152	<0.001	1.927 (1.433, 2.592)
Substance Use Treatment Referral for Alcohol Use	Pass	48	722	0.0665	-0.139	1.044	0.894	0.870 (0.113, 6.700)	0.013	1.058	0.990	1.013 (0.128, 8.023)
	Not eligible				0.570	0.260	0.030	1.769 (1.063, 2.944)	0.102	0.278	0.715	1.107 (0.643, 1.908)
Nicotine Replacement Therapy	Pass	1138	2543	0.4475	0.062	0.242	0.798	1.064 (0.663, 1.708)	0.296	0.254	0.245	1.345 (0.818, 2.212)
	Not eligible				0.510	0.177	0.004	1.665 (1.178, 2.353)	1.238	0.341	<0.001	3.448 (1.771, 6.714)
Polysomnography	Pass	46	5998	0.0077	-	-	-	-	-	-	-	-
	Not eligible				-	-	-	-	-	-	-	-
Neurology Consult	Pass	5329	8052	0.6618	-0.463	0.120	<0.001	0.630 (0.498, 0.796)	-0.395	0.129	0.003	0.674 (0.524, 0.866)
	Not eligible				1.799	0.490	<0.001	6.045 (2.319, 15.759)	1.704	0.538	0.002	5.497 (1.919, 15.749)
Without-Fail		1216	7933	0.1533	-0.211	0.178	0.238	0.810 (0.572, 1.147)	-0.432	0.189	0.024	0.649 (0.449, 0.939)

© 2019 Bravata DM et al. *JAMA Network Open.*

eTable 3. Multivariable Modeling Results Including Patients Ineligible for Processes of Care: 1-Year Mortality

					Univariate Models				Multivariable			
Process	Effect	Pass	Eligible	Pass Rate	Estimate	Std Error	p-value	Odds Ratio (95% Confidence Interval)	Estimate	Std Error	p-value	Odds Ratio (95% Confidence Interval)
Carotid Artery Imaging	Pass	4667	7932	0.5884	-0.508	0.077	<0.001	0.601 (0.518, 0.699)	-0.494	0.083	<0.001	0.610 (0.519, 0.718)
	Not eligible				0.814	0.203	<0.001	2.256 (1.517, 3.356)	0.998	0.229	<0.001	2.712 (1.731, 4.247)
Carotid Stenosis Procedure	Pass	63	279	0.2258	-0.129	0.805	0.873	0.879 (0.182, 4.249)	-0.199	0.830	0.811	0.820 (0.162, 4.152)
	Not eligible				0.936	0.363	0.011	2.548 (1.253, 5.185)	0.702	0.377	0.064	2.019 (0.966, 4.217)
Hypertension Management	Pass	664	2823	0.2352	-0.293	0.169	0.084	0.746 (0.536, 1.038)	-0.195	0.180	0.281	0.823 (0.578, 1.171)
	Not eligible				0.161	0.087	0.064	1.175 (0.992, 1.393)	0.135	0.094	0.152	1.144 (0.952, 1.374)
Hypertension Control	Pass	4666	6139	0.7601	0.031	0.133	0.815	1.032 (0.796, 1.338)	0.041	0.140	0.772	1.041 (0.793, 1.368)
	Not eligible				0.568	0.155	0.000	1.765 (1.304, 2.388)	0.486	0.164	0.003	1.625 (1.180, 2.239)
Antihypertensive Medication Class	Pass	3894	7186	0.5419	-0.459	0.078	<0.001	0.632 (0.542, 0.736)	-0.352	0.086	<0.001	0.703 (0.595, 0.831)
	Not eligible				-0.602	0.139	<0.001	0.548 (0.418, 0.719)	0.154	0.169	0.363	1.166 (0.839, 1.622)
Lipid Measurement	Pass	6334	7933	0.7984	-0.410	0.087	<0.001	0.664 (0.560, 0.787)	-0.440	0.096	<0.001	0.644 (0.534, 0.777)
	Not eligible				0.783	0.210	0.000	2.188 (1.450, 3.301)	0.940	0.238	0.000	2.559 (1.608, 4.073)
Lipid Management	Pass	5001	6174	0.8100	-0.212	0.107	0.049	0.809 (0.657, 0.997)	-0.399	0.120	0.001	0.671 (0.531, 0.848)
	Not eligible				0.163	0.118	0.166	1.178 (0.936, 1.482)	-0.084	0.130	0.518	0.920 (0.714, 1.185)
Discharged on Statin	Pass	4992	6174	0.8086	-0.189	0.107	0.078	0.827 (0.671, 1.020)	-0.363	0.120	0.003	0.695 (0.550, 0.879)
	Not eligible				0.181	0.118	0.125	1.199 (0.952, 1.509)	-0.055	0.130	0.671	0.946 (0.734, 1.219)
Cholesterol Lowering Intensification	Pass	1146	4643	0.2468	-0.897	0.153	<0.001	0.408 (0.302, 0.550)	-0.577	0.161	0.000	0.561 (0.409, 0.770)
	Not eligible				0.111	0.077	0.152	1.117 (0.961, 1.299)	0.228	0.087	0.009	1.256 (1.060, 1.490)
High or Moderate Potency Statin at Discharge	Pass	2251	7279	0.3093	0.376	0.083	<0.001	1.456 (1.238, 1.713)	-0.138	0.095	0.146	0.871 (0.724, 1.048)
	Not eligible				0.629	0.113	<0.001	1.875 (1.504, 2.337)	0.412	0.126	0.001	1.510 (1.180, 1.933)
Brain Imaging	Pass	6795	7849	0.8657	-0.158	0.108	0.145	0.854 (0.692, 1.054)	-0.167	0.122	0.173	0.846 (0.666, 1.074)
	Not eligible				0.718	0.196	0.000	2.051 (1.399, 3.006)	0.676	0.221	0.003	1.966 (1.276, 3.028)
Electrocardiography	Pass	5196	7851	0.6618	-0.096	0.083	0.250	0.909 (0.773, 1.069)	-0.176	0.088	0.048	0.838 (0.705, 0.997)
	Not eligible				0.801	0.182	<0.001	2.228 (1.562, 3.177)	0.725	0.204	0.001	2.065 (1.386, 3.076)
Telemetry	Pass	2417	4932	0.4901	-0.042	0.105	0.687	0.958 (0.780, 1.178)	0.011	0.109	0.919	1.011 (0.817, 1.252)
	Not eligible				0.349	0.090	0.000	1.417 (1.188, 1.691)	0.145	0.111	0.193	1.156 (0.930, 1.438)
Holter	Pass	341	6707	0.0508	-0.469	0.270	0.084	0.626 (0.369, 1.061)	-0.279	0.276	0.314	0.757 (0.441, 1.299)
	Not eligible				1.018	0.103	<0.001	2.768 (2.265, 3.382)	0.134	0.232	0.564	1.143 (0.727, 1.799)

© 2019 Bravata DM et al. *JAMA Network Open.*

eTable 3. Multivariable Modeling Results Including Patients Ineligible for Processes of Care: 1-Year Mortality (continued)

					Univariate Models				Multivariable			
Process	Effect	Pass	Eligible	Pass Rate	Estimate	Std Error	p-value	Odds Ratio (95% Confidence Interval)	Estimate	Std Error	p-value	Odds Ratio (95% Confidence Interval)
Antithrombotics by Hospital Day 2	Pass	6539	7645	0.8553	-0.121	0.107	0.259	0.886 (0.719, 1.092)	-0.370	0.118	0.002	0.691 (0.549, 0.869)
	Not eligible				0.445	0.165	0.008	1.561 (1.130, 2.157)	0.096	0.183	0.600	1.101 (0.770, 1.573)
Antithrombotics at Discharge	Pass	6584	7599	0.8664	-0.084	0.113	0.458	0.920 (0.737, 1.147)	-0.375	0.124	0.003	0.687 (0.539, 0.876)
	Not eligible				0.722	0.158	<0.001	2.058 (1.512, 2.801)	0.360	0.175	0.041	1.434 (1.018, 2.019)
Anticoagulation for Atrial Fibrillation	Pass	829	1071	0.7740	-0.384	0.176	0.030	0.681 (0.483, 0.961)	-0.537	0.192	0.006	0.585 (0.402, 0.850)
	Not eligible				-1.228	0.157	<0.001	0.293 (0.216, 0.398)	-0.300	0.216	0.166	0.741 (0.486, 1.130)
INR Measured	Pass	466	486	0.9589	0.254	0.762	0.740	1.289 (0.290, 5.720)	0.342	0.819	0.677	1.407 (0.283, 6.986)
	Not eligible				-0.365	0.751	0.628	0.694 (0.160, 3.016)	0.558	0.813	0.494	1.747 (0.356, 8.563)
Anticoagulation Quality	Pass	239	466	0.5129	-0.431	0.279	0.125	0.650 (0.377, 1.122)	-0.576	0.294	0.052	0.562 (0.316, 1.000)
	Not eligible				-0.823	0.191	<0.001	0.439 (0.302, 0.637)	-0.078	0.221	0.723	0.925 (0.600, 1.425)
HbA1c Measurement	Pass	2593	3297	0.7865	-0.244	0.132	0.065	0.783 (0.605, 1.013)	-0.186	0.144	0.197	0.830 (0.627, 1.100)
	Not eligible				-0.294	0.124	0.019	0.745 (0.585, 0.951)	0.255	0.185	0.169	1.291 (0.899, 1.853)
Hypoglycemic Medication	Pass	195	571	0.3415	-0.047	0.399	0.907	0.954 (0.438, 2.081)	0.366	0.412	0.376	1.442 (0.644, 3.230)
	Not eligible				0.447	0.234	0.058	1.563 (0.988, 2.473)	0.439	0.249	0.080	1.551 (0.953, 2.523)
Deep Vein Thrombosis (DVT) Prophylaxis	Pass	3451	4294	0.8037	-0.063	0.120	0.603	0.939 (0.743, 1.188)	-0.174	0.129	0.179	0.840 (0.653, 1.081)
	Not eligible				-0.397	0.122	0.001	0.672 (0.530, 0.854)	-0.164	0.132	0.216	0.849 (0.655, 1.099)
Rehabilitation Needs Assessment	Pass	3744	7671	0.4881	0.406	0.081	<0.001	1.501 (1.280, 1.761)	0.053	0.091	0.563	1.054 (0.882, 1.261)
	Not eligible				0.596	0.261	0.023	1.814 (1.090, 3.021)	0.689	0.284	0.016	1.991 (1.143, 3.470)
Speech-Language Pathology Consultation	Pass	2068	5653	0.3658	0.385	0.091	<0.001	1.469 (1.230, 1.756)	0.065	0.103	0.529	1.067 (0.873, 1.304)
	Not eligible				0.191	0.090	0.036	1.210 (1.014, 1.443)	0.372	0.098	0.000	1.451 (1.197, 1.759)
Substance Use Treatment Referral for Alcohol Use	Pass	48	722	0.0665	-0.187	0.615	0.761	0.829 (0.249, 2.763)	-0.036	0.638	0.955	0.965 (0.277, 3.358)
	Not eligible				0.363	0.152	0.019	1.438 (1.067, 1.937)	-0.124	0.167	0.461	0.884 (0.637, 1.226)
Nicotine Replacement Therapy	Pass	1138	2543	0.4475	-0.170	0.153	0.268	0.844 (0.626, 1.138)	0.073	0.162	0.654	1.075 (0.783, 1.476)
	Not eligible				0.339	0.107	0.002	1.403 (1.137, 1.731)	0.574	0.259	0.028	1.776 (1.069, 2.949)
Polysomnography	Pass	46	5998	0.0077	-	-	-	-	-	-	-	-
	Not eligible				-0.089	0.130	0.497	0.915 (0.709, 1.181)	0.007	0.142	0.959	1.007 (0.764, 1.329)
Neurology Consult	Pass	5329	8052	0.6618	-0.368	0.078	<0.001	0.692 (0.594, 0.806)	-0.303	0.084	0.000	0.739 (0.627, 0.871)
	Not eligible				1.646	0.423	0.000	5.186 (2.268, 11.857)	1.508	0.473	0.002	4.516 (1.792, 11.382)
Without-Fail		1216	7933	0.1533	-0.129	0.110	0.241	0.879 (0.710, 1.089)	-0.374	0.118	0.002	0.688 (0.546, 0.867)

© 2019 Bravata DM et al. *JAMA Network Open.*

eTable 4. Multivariable Modeling Results Including Patients Ineligible for Processes of Care: 90-Day Recurrent Stroke

					Univariate Models				Multivariable			
Process	Effect	Pass	Eligible	Pass Rate	Estimate	Std Error	p-value	Odds Ratio (95% Confidence Interval)	Estimat e	Std Error	p-value	Odds Ratio (95% Confidence Interval)
Carotid Artery Imaging	Pass	4569	7683	0.5947	-0.082	0.098	0.402	0.921 (0.760, 1.116)	-0.116	0.101	0.253	0.891 (0.731, 1.085)
	Not eligible				0.788	0.288	0.007	2.199 (1.253, 3.861)	0.764	0.296	0.011	2.146 (1.204, 3.828)
Carotid Stenosis Procedure	Pass	63	279	0.2258	-1.535	0.747	0.042	0.215 (0.050, 0.928)	-1.721	0.764	0.026	0.179 (0.040, 0.796)
	Not eligible				-1.684	0.215	<0.001	0.186 (0.122, 0.283)	-1.743	0.227	<0.001	0.175 (0.112, 0.273)
Hypertension Management	Pass	657	2763	0.2378	-0.002	0.174	0.989	0.998 (0.710, 1.401)	-0.027	0.178	0.880	0.974 (0.688, 1.378)
	Not eligible				-0.285	0.105	0.007	0.752 (0.612, 0.924)	-0.307	0.107	0.005	0.736 (0.596, 0.907)
Hypertension Control	Pass	4666	6139	0.7601	-	-	-	-	-	-	-	-
	Not eligible				-	-	-	-	-	-	-	-
Antihypertensive Medication Class	Pass	3797	6928	0.5481	-0.291	0.100	0.004	0.748 (0.614, 0.910)	-0.303	0.104	0.004	0.739 (0.603, 0.906)
	Not eligible				-0.246	0.163	0.132	0.782 (0.568, 1.075)	-0.112	0.190	0.555	0.894 (0.617, 1.296)
Lipid Measurement	Pass	6156	7684	0.8012	-0.189	0.115	0.103	0.827 (0.660, 1.037)	-0.248	0.119	0.038	0.780 (0.618, 0.985)
	Not eligible				0.697	0.296	0.020	2.008 (1.125, 3.582)	0.649	0.304	0.034	1.913 (1.055, 3.469)
Lipid Management	Pass	4880	6008	0.8123	-0.163	0.132	0.217	0.850 (0.657, 1.099)	-0.312	0.139	0.026	0.732 (0.558, 0.961)
	Not eligible				-0.223	0.155	0.153	0.800 (0.591, 1.085)	-0.330	0.160	0.040	0.719 (0.526, 0.983)
Discharged on Statin	Pass	4870	6008	0.8106	0.050	0.139	0.721	1.051 (0.801, 1.379)	-0.075	0.146	0.608	0.928 (0.698, 1.234)
	Not eligible				-0.052	0.162	0.750	0.950 (0.692, 1.303)	-0.142	0.166	0.393	0.868 (0.627, 1.200)
Cholesterol Lowering Intensification	Pass	1135	4533	0.2504	0.006	0.145	0.969	1.006 (0.757, 1.335)	0.007	0.152	0.964	1.007 (0.748, 1.355)
	Not eligible				0.041	0.103	0.694	1.041 (0.852, 1.273)	0.095	0.108	0.381	1.099 (0.890, 1.358)
High or Moderate Potency Statin at Discharge	Pass	2168	7071	0.3066	-0.248	0.114	0.031	0.780 (0.624, 0.976)	-0.318	0.123	0.010	0.727 (0.572, 0.924)
	Not eligible				0.048	0.159	0.764	1.049 (0.768, 1.433)	-0.004	0.165	0.983	0.996 (0.721, 1.377)
Brain Imaging	Pass	6587	7606	0.8660	-0.111	0.137	0.420	0.895 (0.684, 1.171)	0.098	0.147	0.505	1.104 (0.827, 1.472)
	Not eligible				0.439	0.274	0.111	1.550 (0.907, 2.650)	0.479	0.283	0.092	1.614 (0.928, 2.805)
Electrocardiography	Pass	5041	7608	0.6626	0.047	0.105	0.655	1.048 (0.854, 1.287)	0.120	0.108	0.269	1.127 (0.912, 1.393)
	Not eligible				0.578	0.258	0.026	1.782 (1.076, 2.949)	0.486	0.264	0.067	1.626 (0.971, 2.725)
Telemetry	Pass	2354	4793	0.4911	0.209	0.128	0.105	1.232 (0.959, 1.583)	0.201	0.129	0.122	1.222 (0.949, 1.574)
	Not eligible				0.311	0.118	0.009	1.365 (1.083, 1.719)	0.336	0.131	0.011	1.400 (1.084, 1.808)
Holter	Pass	341	6707	0.0508	-0.588	0.511	0.250	0.555 (0.205, 1.507)	-0.491	0.512	0.339	0.612 (0.225, 1.666)
	Not eligible				0.183	0.233	0.434	1.201 (0.761, 1.894)	-0.977	0.419	0.021	0.376 (0.166, 0.854)

© 2019 Bravata DM et al. *JAMA Network Open.*

eTable 4. Multivariable Modeling Results Including Patients Ineligible for Processes of Care: 90-Day Recurrent Stroke (continued)

					Univariate Models				Multivariable			
Process	Effect	Pass	Eligible	Pass Rate	Estimate	Std Error	p-value	Odds Ratio (95% Confidence Interval)	Estimat e	Std Error	p-value	Odds Ratio (95% Confidence Interval)
Antithrombotics by Hospital Day 2	Pass	6347	7408	0.8568	-0.034	0.139	0.808	0.967 (0.737, 1.269)	-0.089	0.144	0.537	0.915 (0.691, 1.212)
	Not eligible				0.343	0.223	0.125	1.410 (0.911, 2.181)	0.332	0.228	0.147	1.394 (0.892, 2.179)
Antithrombotics at Discharge	Pass	6404	7384	0.8673	-0.061	0.143	0.671	0.941 (0.712, 1.244)	-0.143	0.148	0.336	0.867 (0.649, 1.158)
	Not eligible				0.387	0.218	0.077	1.473 (0.962, 2.255)	0.343	0.223	0.127	1.409 (0.910, 2.181)
Anticoagulation for Atrial Fibrillation	Pass	777	996	0.7801	-0.316	0.296	0.288	0.729 (0.408, 1.302)	-0.357	0.301	0.237	0.700 (0.388, 1.261)
	Not eligible				-0.272	0.258	0.294	0.762 (0.460, 1.263)	0.065	0.337	0.847	1.067 (0.552, 2.062)
INR Measured	Pass	466	486	0.9589	-1.373	1.105	0.217	0.253 (0.029, 2.203)	-1.408	1.119	0.211	0.245 (0.027, 2.185)
	Not eligible				-0.865	1.030	0.403	0.421 (0.056, 3.155)	-0.339	1.058	0.749	0.713 (0.090, 5.639)
Anticoagulation Quality	Pass	239	466	0.5129	-0.755	0.871	0.387	0.470 (0.086, 2.583)	-0.723	0.869	0.407	0.485 (0.089, 2.657)
	Not eligible				0.193	0.511	0.706	1.213 (0.446, 3.297)	0.769	0.544	0.159	2.157 (0.745, 6.244)
HbA1c Measurement	Pass	2512	3187	0.7882	0.219	0.187	0.243	1.245 (0.863, 1.796)	0.216	0.190	0.256	1.241 (0.856, 1.799)
	Not eligible				0.065	0.181	0.719	1.067 (0.749, 1.520)	0.484	0.241	0.046	1.623 (1.013, 2.598)
Hypoglycemic Medication	Pass	195	571	0.3415	-0.172	0.608	0.778	0.842 (0.257, 2.764)	-0.144	0.613	0.815	0.866 (0.261, 2.874)
	Not eligible				-0.143	0.348	0.681	0.867 (0.439, 1.711)	-0.248	0.365	0.498	0.781 (0.382, 1.594)
Deep Vein Thrombosis (DVT) Prophylaxis	Pass	3317	4117	0.8057	0.545	0.191	0.005	1.724 (1.186, 2.506)	0.533	0.193	0.006	1.704 (1.168, 2.486)
	Not eligible				0.336	0.192	0.082	1.399 (0.960, 2.038)	0.448	0.196	0.023	1.566 (1.068, 2.295)
Rehabilitation Needs Assessment	Pass	3744	7671	0.4881	0.354	0.123	0.004	1.424 (1.120, 1.811)	0.146	0.133	0.272	1.157 (0.893, 1.500)
	Not eligible				-0.348	0.591	0.557	0.706 (0.222, 2.245)	-0.402	0.596	0.501	0.669 (0.209, 2.145)
Speech-Language Pathology Consultation	Pass	1974	5474	0.3606	0.321	0.119	0.007	1.378 (1.093, 1.738)	0.045	0.126	0.720	1.046 (0.817, 1.340)
	Not eligible				0.336	0.113	0.003	1.399 (1.122, 1.744)	0.293	0.117	0.013	1.340 (1.066, 1.683)
Substance Use Treatment Referral for Alcohol Use	Pass	47	708	0.0664	0.529	0.500	0.292	1.696 (0.638, 4.510)	0.542	0.507	0.287	1.720 (0.638, 4.638)
	Not eligible				-0.117	0.164	0.477	0.890 (0.645, 1.227)	-0.030	0.173	0.864	0.971 (0.692, 1.362)
Nicotine Replacement Therapy	Pass	1110	2483	0.4470	0.030	0.167	0.857	1.031 (0.744, 1.428)	-0.021	0.169	0.903	0.980 (0.704, 1.363)
	Not eligible				-0.050	0.126	0.693	0.951 (0.743, 1.218)	0.198	0.298	0.507	1.219 (0.680, 2.183)
Polysomnography	Pass	46	5998	0.0077	-	-	-	-	-	-	-	-
	Not eligible				-	-	-	-	-	-	-	-
Neurology Consult	Pass	5180	7784	0.6655	-0.052	0.101	0.606	0.949 (0.779, 1.157)	-0.085	0.105	0.419	0.918 (0.748, 1.128)
	Not eligible				-0.129	1.039	0.901	0.879 (0.115, 6.706)	-0.346	1.046	0.741	0.707 (0.091, 5.473)
Without-Fail		1183	7684	0.1540	-0.260	0.146	0.077	0.771 (0.580, 1.025)	-0.308	0.150	0.042	0.735 (0.548, 0.985)

© 2019 Bravata DM et al. *JAMA Network Open.*

eTable 5. Multivariable Modeling Results Including Patients Ineligible for Processes of Care: 1-Year Recurrent Stroke

					Univariate Models				Multivariable			
Process	Effect	Pass	Eligible	Pass Rate	Estimate	Std Error	p-value	Odds Ratio (95% Confidence Interval)	Estimat e	Std Error	p-value	Odds Ratio (95% Confidence Interval)
Carotid Artery Imaging	Pass	4365	7276	0.5999	-0.046	0.078	0.550	0.955 (0.820, 1.111)	-0.064	0.081	0.430	0.938 (0.802, 1.098)
	Not eligible				0.464	0.265	0.082	1.590 (0.947, 2.671)	0.481	0.274	0.081	1.617 (0.947, 2.763)
Carotid Stenosis Procedure	Pass	63	279	0.2258	-2.178	0.737	0.004	0.113 (0.027, 0.479)	-2.164	0.743	0.004	0.115 (0.027, 0.491)
	Not eligible				-1.344	0.171	<0.001	0.261 (0.187, 0.365)	-1.360	0.179	<0.001	0.257 (0.181, 0.364)
Hypertension Management	Pass	630	2625	0.2400	0.029	0.140	0.837	1.029 (0.783, 1.354)	0.036	0.144	0.801	1.037 (0.782, 1.374)
	Not eligible				-0.193	0.084	0.023	0.824 (0.699, 0.972)	-0.211	0.087	0.016	0.810 (0.683, 0.960)
Hypertension Control	Pass	4666	6139	0.7601	-0.474	0.134	0.001	0.622 (0.479, 0.809)	-0.375	0.138	0.007	0.687 (0.525, 0.900)
	Not eligible				-0.249	0.178	0.163	0.780 (0.551, 1.104)	-0.194	0.181	0.284	0.823 (0.578, 1.173)
Antihypertensive Medication Class	Pass	3614	6553	0.5515	-0.171	0.079	0.032	0.843 (0.723, 0.984)	-0.169	0.082	0.041	0.845 (0.719, 0.992)
	Not eligible				-0.447	0.140	0.002	0.640 (0.487, 0.841)	-0.220	0.161	0.174	0.802 (0.585, 1.100)
Lipid Measurement	Pass	5853	7277	0.8043	-0.112	0.094	0.235	0.894 (0.745, 1.074)	-0.169	0.097	0.083	0.844 (0.698, 1.021)
	Not eligible				0.414	0.272	0.129	1.513 (0.889, 2.572)	0.396	0.281	0.160	1.486 (0.859, 2.573)
Lipid Management	Pass	4626	5697	0.8120	-0.005	0.110	0.963	0.995 (0.803, 1.233)	-0.156	0.116	0.182	0.856 (0.682, 1.074)
	Not eligible				0.007	0.126	0.958	1.007 (0.786, 1.289)	-0.107	0.131	0.412	0.898 (0.696, 1.160)
Discharged on Statin	Pass	4618	5697	0.8106	0.155	0.114	0.174	1.168 (0.935, 1.460)	0.027	0.120	0.820	1.028 (0.813, 1.300)
	Not eligible				0.138	0.130	0.289	1.148 (0.890, 1.481)	0.039	0.134	0.769	1.040 (0.800, 1.353)
Cholesterol Lowering Intensification	Pass	1105	4300	0.2570	0.058	0.112	0.605	1.060 (0.851, 1.319)	0.161	0.118	0.173	1.175 (0.933, 1.480)
	Not eligible				0.018	0.082	0.824	1.018 (0.868, 1.195)	0.114	0.087	0.190	1.121 (0.946, 1.328)
High or Moderate Potency Statin at Discharge	Pass	2021	6697	0.3018	-0.127	0.089	0.155	0.881 (0.740, 1.048)	-0.230	0.096	0.018	0.794 (0.658, 0.959)
	Not eligible				0.196	0.123	0.113	1.216 (0.956, 1.548)	0.138	0.129	0.288	1.148 (0.891, 1.477)
Brain Imaging	Pass	6252	7203	0.8680	-0.032	0.112	0.772	0.968 (0.778, 1.205)	0.160	0.121	0.186	1.173 (0.927, 1.486)
	Not eligible				0.255	0.240	0.290	1.290 (0.807, 2.062)	0.322	0.249	0.198	1.380 (0.848, 2.247)
Electrocardiography	Pass	4788	7205	0.6645	0.044	0.082	0.592	1.045 (0.890, 1.228)	0.115	0.086	0.185	1.122 (0.947, 1.328)
	Not eligible				0.325	0.226	0.152	1.384 (0.889, 2.156)	0.273	0.235	0.246	1.314 (0.831, 2.079)
Telemetry	Pass	2251	4568	0.4928	0.037	0.098	0.709	1.037 (0.856, 1.257)	0.040	0.101	0.690	1.041 (0.855, 1.268)
	Not eligible				0.097	0.091	0.288	1.102 (0.922, 1.318)	0.073	0.103	0.482	1.075 (0.879, 1.315)
Holter	Pass	341	6707	0.0508	-0.838	0.325	0.011	0.433 (0.229, 0.818)	-0.722	0.327	0.028	0.486 (0.256, 0.922)
	Not eligible				0.183	0.142	0.198	1.201 (0.910, 1.586)	-0.488	0.294	0.098	0.614 (0.346, 1.090)

© 2019 Bravata DM et al. *JAMA Network Open.*

eTable 5. Multivariable Modeling Results Including Patients Ineligible for Processes of Care: 1-Year Recurrent Stroke (continued)

					Univariate Models				Multivariable			
Process	Effect	Pass	Eligible	Pass Rate	Estimate	Std Error	p-value	Odds Ratio (95% Confidence Interval)	Estimate	Std Error	p-value	Odds Ratio (95% Confidence Interval)
Antithrombotics by Hospital Day 2	Pass	6007	7014	0.8564	0.088	0.114	0.440	1.092 (0.874, 1.365)	0.015	0.119	0.898	1.015 (0.805, 1.280)
	Not eligible				0.465	0.181	0.011	1.592 (1.118, 2.267)	0.403	0.187	0.032	1.497 (1.039, 2.157)
Antithrombotics at Discharge	Pass	6059	6990	0.8668	0.104	0.119	0.381	1.110 (0.880, 1.400)	0.003	0.123	0.980	1.003 (0.788, 1.277)
	Not eligible				0.513	0.179	0.005	1.670 (1.176, 2.371)	0.431	0.185	0.021	1.539 (1.072, 2.209)
Anticoagulation for Atrial Fibrillation	Pass	703	896	0.7846	-0.367	0.239	0.126	0.693 (0.434, 1.105)	-0.400	0.245	0.104	0.670 (0.415, 1.082)
	Not eligible				-0.356	0.209	0.090	0.700 (0.466, 1.054)	-0.019	0.273	0.945	0.981 (0.575, 1.674)
INR Measured	Pass	466	486	0.9589	0.058	1.050	0.956	1.060 (0.136, 8.272)	0.060	1.071	0.955	1.062 (0.131, 8.637)
	Not eligible				0.218	1.030	0.833	1.244 (0.166, 9.332)	0.560	1.057	0.597	1.751 (0.221, 13.849)
Anticoagulation Quality	Pass	239	466	0.5129	0.259	0.427	0.544	1.296 (0.563, 2.984)	0.178	0.432	0.681	1.195 (0.513, 2.784)
	Not eligible				0.303	0.328	0.358	1.353 (0.712, 2.572)	0.590	0.353	0.097	1.804 (0.904, 3.602)
HbA1c Measurement	Pass	2374	3001	0.7911	0.170	0.142	0.232	1.185 (0.898, 1.565)	0.151	0.145	0.300	1.163 (0.875, 1.545)
	Not eligible				-0.151	0.138	0.275	0.860 (0.657, 1.126)	0.120	0.191	0.533	1.127 (0.775, 1.638)
Hypoglycemic Medication	Pass	195	571	0.3415	0.388	0.334	0.246	1.474 (0.768, 2.830)	0.452	0.339	0.185	1.571 (0.809, 3.050)
	Not eligible				0.046	0.221	0.834	1.047 (0.680, 1.614)	0.079	0.231	0.734	1.082 (0.688, 1.700)
Deep Vein Thrombosis (DVT) Prophylaxis	Pass	3125	3880	0.8054	0.219	0.134	0.105	1.244 (0.957, 1.617)	0.187	0.137	0.172	1.206 (0.923, 1.576)
	Not eligible				-0.026	0.135	0.846	0.974 (0.748, 1.268)	0.065	0.139	0.641	1.067 (0.813, 1.399)
Rehabilitation Needs Assessment	Pass	3744	7671	0.4881	0.377	0.086	<0.001	1.457 (1.231, 1.726)	0.173	0.094	0.066	1.189 (0.990, 1.428)
	Not eligible				-0.108	0.371	0.771	0.897 (0.434, 1.854)	-0.142	0.376	0.706	0.868 (0.416, 1.809)
Speech-Language Pathology Consultation	Pass	1856	5181	0.3582	0.303	0.092	0.001	1.354 (1.131, 1.621)	0.101	0.099	0.305	1.107 (0.912, 1.342)
	Not eligible				0.144	0.090	0.113	1.155 (0.967, 1.378)	0.122	0.094	0.199	1.129 (0.939, 1.358)
Substance Use Treatment Referral for Alcohol Use	Pass	47	681	0.0690	0.438	0.408	0.285	1.550 (0.697, 3.445)	0.489	0.418	0.244	1.630 (0.720, 3.689)
	Not eligible				-0.127	0.129	0.330	0.881 (0.684, 1.135)	-0.101	0.138	0.463	0.904 (0.691, 1.183)
Nicotine Replacement Therapy	Pass	1069	2375	0.4501	0.094	0.133	0.481	1.099 (0.846, 1.426)	0.073	0.136	0.593	1.075 (0.824, 1.404)
	Not eligible				0.021	0.102	0.836	1.021 (0.837, 1.246)	0.097	0.257	0.708	1.102 (0.666, 1.822)
Polysomnography	Pass	46	5998	0.0077	-0.071	0.726	0.922	0.931 (0.225, 3.852)	0.090	0.733	0.903	1.094 (0.261, 4.583)
	Not eligible				-0.045	0.151	0.764	0.956 (0.712, 1.283)	-0.121	0.159	0.445	0.886 (0.649, 1.208)
Neurology Consult	Pass	4938	7373	0.6697	-0.020	0.081	0.806	0.980 (0.837, 1.148)	-0.024	0.084	0.773	0.976 (0.827, 1.151)
	Not eligible				-0.456	1.043	0.663	0.634 (0.082, 4.872)	-0.868	1.064	0.416	0.420 (0.052, 3.363)
Without-Fail		1124	7277	0.1545	-0.146	0.110	0.186	0.864 (0.697, 1.071)	-0.211	0.114	0.067	0.810 (0.649, 1.012)

© 2019 Bravata DM et al. *JAMA Network Open.*

eTable 6. Sensitivity Analyses: Adjusted Association of Without-Fail Rate With Outcomes

Without-Fail Rate*	90-Day Mortality		1-Year Mortality		90-Day Recurrent Ischemic Stroke		1-Y[illegible]
	aOR (95% CI)	P-Value	aOR (95% CI)	P-Value	aOR (95% CI)	P-Value	[illegible]
Overall	0.649 (0.449, 0.939)	0.024	0.688 (0.546, 0.867)	0.002	0.735 (0.548, 0.985)	0.042	0.81[illegible]
Age < 65 years	0.295 (0.088, 0.992)	0.052	0.558 (0.299, 1.038)	0.069	0.478 (0.271, 0.841)	0.012	0.56[illegible]
Age ≥65 years	0.709 (0.477, 1.053)	0.092	0.707 (0.549, 0.910)	0.008	0.910 (0.643, 1.290)	0.599	0.98[illegible]
Transient ischemic attack	0.642 (0.336, 1.225)	0.183	0.676 (0.471, 0.969)	0.036	0.804 (0.489, 1.320)	0.391	0.86[illegible]
Non-severe stroke	0.645 (0.411, 1.014)	0.061	0.677 (0.499, 0.921)	0.015	0.692 (0.480, 0.996)	0.051	0.76[illegible]

*The without-fail measure consisted of six processes of care: carotid artery imaging, hypertension medication intensification, high or moderate potency statin, brain imaging, antithrombotics at discharge, and anticoagulation for atrial fibrillation. A patient passed the without-fail rate if they received all of the processes for which they were eligible.

**Each adjusted odds ratio (aOR) was generated from a multivariable model that included all of the covariates listed in Table 2.

© 2019 Bravata DM et al. *JAMA Network Open.*

eTable 7. Sensitivity Analyses: Adjusted Analyses Between Medication Categories and Outcomes

Processes of Care	90-Day Mortality		1-Year Mortality		90-Day Recurrent Ischemic Stroke	
	aOR (95% CI)	P-Value	aOR (95% CI)	P-Value	aOR (95% CI)	P-Value
Statins at discharge						
Did not receive medication either before or after TIA	reference	-	reference	-	reference	-
Original definition*	0.514 (0.364, 0.726)	<0.001	0.695 (0.550, 0.879)	0.003	0.928 (0.698, 1.234)	0.608
Prior use	0.503 (0.349, 0.725)	<0.001	0.738 (0.576, 0.944)	0.016	0.868 (0.641, 1.177)	0.366
New medication post-TIA	0.539 (0.348, 0.834)	0.006	0.611 (0.456, 0.819)	0.001	1.018 (0.737, 1.406)	0.916
Antithrombotics at discharge						
Did not receive medication either before or after TIA	reference	-	reference	-	reference	-
Original definition*	0.593 (0.412, 0.856)	0.006	0.687 (0.539, 0.876)	0.003	0.867 (0.649, 1.158)	0.336
Prior use	0.588 (0.395, 0.877)	0.010	0.693 (0.532, 0.902)	0.007	0.790 (0.572, 1.092)	0.155
New medication post-TIA	0.599 (0.404, 0.888)	0.011	0.683 (0.527, 0.885)	0.004	0.922 (0.680, 1.249)	0.602
Anticoagulation for atrial fibrillation						
Did not receive medication either before or after TIA	reference	-	reference	-	reference	-
Original definition*	0.593 (0.353, 0.994)	0.049	0.585 (0.402, 0.850)	0.006	0.700 (0.388, 1.261)	0.237
Prior use	0.586 (0.331, 1.038)	0.069	0.568 (0.375, 0.860)	0.008	0.572 (0.294, 1.113)	0.102
New medication post-TIA	0.608 (0.333, 1.110)	0.107	0.605 (0.393, 0.932)	0.024	0.888 (0.456, 1.729)	0.727

*The original definition is the one that was used for the main analysis and those results are provided in Table 3.

© 2019 Bravata DM et al. *JAMA Network Open.*

eTable 8. Adjusted P Values Based on the False Discovery Rate

	90 Day Mortality		365 Day Mortality		90 Day Stroke		365 Day Stroke	
Effect	Raw	FDR	Raw	FDR	Raw	FDR	Raw	FDR
Carotid Artery Imaging	<0.0001	<0.0001	<0.0001	<0.0001	0.2527	0.5160	0.4302	0.6567
Carotid Stenosis Procedure	-	-	0.8108	0.8709	0.0259	0.1398	0.0042	0.1005
Hypertension medication intensification	0.0805	0.1676	0.2812	0.4531	0.8800	0.9377	0.8010	0.9512
Hypertension Control	-	-	0.7718	0.8608	-	-	0.0069	0.1005
Antihypertensive Medication Class	<0.0001	0.0003	<0.0001	0.0005	0.0040	0.0839	0.0412	0.2389
Lipid Measurement	0.0080	0.0250	<0.0001	0.0001	0.0384	0.1612	0.0834	0.3022
Lipid Management	<0.0001	0.0002	0.0010	0.0049	0.0259	0.1398	0.1820	0.3604
Discharged on Statin	0.0002	0.0013	0.0027	0.0082	0.6083	0.7466	0.8200	0.9512
Cholesterol Lowering Intensification	0.0096	0.0268	0.0004	0.0025	0.9635	0.9635	0.1731	0.3604
High/Moderate Potency Statin	0.2241	0.3753	0.1456	0.3016	0.0100	0.0899	0.0177	0.1707
Hypoglycemic medication intensification	0.2964	0.4359	0.3758	0.5450	0.8145	0.9163	0.1845	0.3604
HbA1c Measurement	0.7486	0.8912	0.1968	0.3357	0.2559	0.5160	0.3002	0.4921
Electrocardiography	0.5392	0.7095	0.0475	0.1147	0.2692	0.5160	0.1850	0.3604
Telemetry	0.8989	0.9771	0.9187	0.9515	0.1222	0.4126	0.6895	0.9089
Holter	0.9466	0.9861	0.3140	0.4792	0.3385	0.5376	0.0284	0.2061
Antithrombotics by Day 2	0.0010	0.0048	0.0019	0.0073	0.5371	0.6906	0.8978	0.9696
Antithrombotics at Discharge	0.0057	0.0204	0.0028	0.0082	0.3357	0.5376	0.9795	0.9795
Anticoagulation for Atrial Fibrillation	0.0493	0.1120	0.0056	0.0147	0.2371	0.5160	0.1040	0.3351
INR Measurement§	-	-	0.6773	0.7857	0.2108	0.5160	0.9552	0.9795
Anticoagulation Quality	0.8822	0.9771	0.0519	0.1157	0.4067	0.5951	0.6805	0.9089
Brain Imaging	0.6075	0.7593	0.1726	0.3237	0.5046	0.6812	0.1864	0.3604
Deep Vein Thrombosis (DVT) Prophylaxis	0.2222	0.3753	0.1786	0.3237	0.0062	0.0839	0.1715	0.3604
Rehabilitation Needs Assessment	0.4259	0.5916	0.5628	0.7096	0.2722	0.5160	0.0655	0.2763
Speech-language Pathology Consultation	0.2252	0.3753	0.5287	0.6969	0.7198	0.8450	0.3054	0.4921
Substance Use Treatment Referral for Alcohol Use	0.9902	0.9902	0.9552	0.9552	0.2866	0.5160	0.2436	0.4416
Nicotine Replacement Therapy	0.2452	0.3831	0.6540	0.7857	0.9029	0.9377	0.5933	0.8602
Polysomnography	-	-	0.4297	0.5933	-	-	0.9028	0.9696
Neurology Consultation	0.0025	0.0106	0.0004	0.0025	0.4188	0.5951	0.7731	0.9512
Without-Fail	0.0238	0.0595	0.0020	0.0073	0.0418	0.1612	0.0667	0.2763

© 2019 Bravata DM et al. *JAMA Network Open.*
